# Complete loss of H3K9 methylation dissolves mouse heterochromatin organization

**DOI:** 10.1038/s41467-021-24532-8

**Published:** 2021-07-16

**Authors:** Thomas Montavon, Nicholas Shukeir, Galina Erikson, Bettina Engist, Megumi Onishi-Seebacher, Devon Ryan, Yaarub Musa, Gerhard Mittler, Alexandra Graff Meyer, Christel Genoud, Thomas Jenuwein

**Affiliations:** 1 Max-Planck Institute of Immunobiology and Epigenetics, Freiburg, Germany; 2 Friedrich Miescher Institute for Biomedical Research, Basel, Switzerland; 3 Present Address: Novartis Institute for Biomedical Research (NIBR), Basel, Switzerland; 4 Present Address: Genedata AG, Basel, Switzerland; 5 Present Address: Thermo Fisher Scientific GmbH, Dreieich, Germany

**Keywords:** Transferases, Gene silencing

## Abstract

Histone H3 lysine 9 (H3K9) methylation is a central epigenetic modification that defines heterochromatin from unicellular to multicellular organisms. In mammalian cells, H3K9 methylation can be catalyzed by at least six distinct SET domain enzymes: Suv39h1/Suv39h2, Eset1/Eset2 and G9a/Glp. We used mouse embryonic fibroblasts (MEFs) with a conditional mutation for *Eset1* and introduced progressive deletions for the other SET domain genes by CRISPR/Cas9 technology. Compound mutant MEFs for all six SET domain lysine methyltransferase (KMT) genes lack all H3K9 methylation states, derepress nearly all families of repeat elements and display genomic instabilities. Strikingly, the 6KO H3K9 KMT MEF cells no longer maintain heterochromatin organization and have lost electron-dense heterochromatin. This is a compelling analysis of H3K9 methylation-deficient mammalian chromatin and reveals a definitive function for H3K9 methylation in protecting heterochromatin organization and genome integrity.

## Abstract (web summary)

Histone H3K9 methylation (H3K9me) states define repressed chromatin in eukaryotic cells. Here the authors reveal complete loss of all H3K9me in mammalian cells through successive deletion of H3K9 methyltransferase genes that results in the dissolution of heterochromatin and the derepression of nearly all repeat families.

## Introduction

Heterochromatin is gene-poor, repeat-rich regions of the genome that are classically defined to remain condensed during interphase^1^. From these early cytological studies, DAPI staining of A/T-rich repeats to high-resolution microscopy, heterochromatin can be described as an electron-dense chromatin material that shows the concentration in subnuclear compartments and is often associated with the nuclear periphery. Heterochromatin has important functions for chromosome segregation, genome integrity, and gene regulation^2,3^.

The methylation of histone H3 at lysine 9 (H3K9) is a hallmark of heterochromatin. It is established by three distinct enzymatic systems that target different regions of the genome. Suv39h enzymes (Suv39h1 and Suv39h2 in mammals) establish H3K9 trimethylation (H3K9me3) at constitutive heterochromatin^4,5^. Setdb1/Eset1 is required for retrotransposon silencing^6,7^. G9a and Glp are predominantly mediating H3K9 dimethylation (H3K9me2) and gene repression at euchromatic loci^8,9^.

A biochemical pathway for heterochromatin formation involves Suv39h-mediated H3K9me3, which is bound by the heterochromatin protein HP1^4,10,11^. HP1 binding promotes compaction and transcriptional repression and stabilizes Suv39h binding to heterochromatin. However, while disruption of Suv39h enzymes results in loss of H3K9me3 and dispersion of HP1, the overall organization of heterochromatin in DAPI-dense foci remains largely intact^5^, indicating that other mechanisms are involved.

Several observations suggest a partial redundancy between H3K9 methylation systems. A sub-stoichiometric complex containing G9a, Suv39h, and Eset1 has been described^12^. *Eset1* knockdown in *Suv39h* double-null MEFs results in partial dispersion of DAPI-dense heterochromatic foci^13^. In *C. elegans*, double deletion of MET-2 (a Setdb1 homolog) and SET-25 (a KMT with some homology to G9a and Suv39h) results in loss of heterochromatin anchoring to the nuclear lamina^14^. Interestingly, *MET-2/SET-25* mutant worms are viable despite lacking all H3K9 methylation, but derepress transposons and satellite repeats, leading to genome instability and sterility^15,16^.

We addressed potential redundancy between H3K9 methylation systems in mouse fibroblast cells by generating compound mutants for all known SET-domain H3K9 lysine methyltransferases (KMT) using the CRISPR/Cas9 technology. We isolated mouse embryonic fibroblast (MEF) cell lines lacking progressively 2, 4, or all 6 H3K9 KMTs. Compound mutant MEF cells lacking all six enzymes lost all H3K9 methylation, derepress multiple families of repeat elements, and display DNA damage and cell death. While mutants retaining the function of some of the H3K9-methylating enzymes maintain heterochromatin clustering, 6KO MEF cells show the complete collapse of heterochromatin foci and loss of electron-dense heterochromatin. These results illustrate the functional redundancy between the three H3K9 KMT systems and provide compelling evidence for an essential function of H3K9 methylation in heterochromatin organization.

## Results

### Gene pair disruptions identify distinct functions for the Suv39h, Eset, and G9a enzymes in directing H3K9 methylation states

**Fig. 1:**
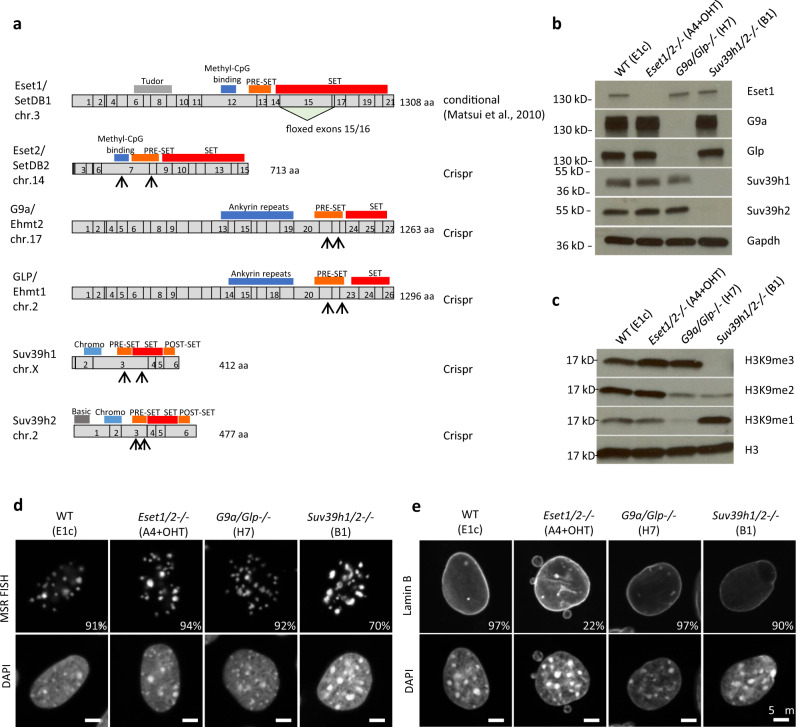
Three distinct gene pairs encode H3K9 KMT enzymes in the mouse genome. **a** Domain structure of the six SET-domain H3K9 KMT. For each gene, numbered boxes indicate exons, and conserved protein domains are indicated above the gene structure. The triangle below *Eset1* illustrates the floxed exons deleted upon tamoxifen treatment. For the other SET-domain H3K9 KMT genes, disruptions were generated by CRISPR/Cas9 technology, with the location of the gRNAs indicated by arrows below the gene structure. **b** Gene pair mutants lacking either *Eset1/Eset2*, *G9a/Glp*, or *Suv39h1/Suv39h2*. Western blots were performed on whole-cell extracts of the mutant clones (top) using specific antibodies indicated on the right. Gapdh was used as a loading control. *Eset1* deletion was probed with extracts after 2 days of induction in culture with 4-hyroxytamoxifen (4-OHT, 1 μM) followed by 2 days in a normal medium. Representative results of two independent experiments. **c** Western blot analysis of H3K9 methylation states in H3K9 KMT gene pair mutants. Acid-extracted histones were probed with antibodies specific for H3K9me1, H3K9me2, and H3K9me3. Total H3 was used as a loading control. Representative results of three independent experiments. Source data are provided as a Source Data file. **d** DNA FISH for major satellite repeats (MSR) in control and H3K9 KMT gene pair mutants. Percentages indicate cells that display the shown pattern. *N* = 325 cells examined for WT, 329 for *Eset1/2−/−*, 301 for *G9a/Glp−/−*, and 384 for *Suv39h1/2−/−* over two independent experiments. For each image, lower panels show DAPI counterstaining of the same nuclei. **e** Immunofluorescence for Lamin B, marking the nuclear lamina. Percentages indicate cells that display the shown pattern. *N* = 402 cells examined for WT, 439 for *Eset1/2−/−*, 321 for *G9a/Glp−/*−, and 410 for *Suv39h1/2−/−* over two independent experiments. For each image, lower panels show DAPI counterstaining of the same nuclei. Scale bars represent 5 μm.

In mouse cells, 6 genes encode SET-domain-containing enzymes with a documented methyltransferase activity toward H3K9: *Suv39h1* and *Suv39h2*, *Eset1/Setdb1* and *Eset2/Setdb2*, and *G9a/Ehmt2* and *Glp/Ehmt1* (Fig. 1a). Since *Setdb1/Eset1*-deficient cells display growth defects, we took advantage of existing mouse embryonic fibroblasts (MEFs) with a tamoxifen-inducible conditional deletion of *Eset1*^6^, hereafter called *Eset1cKO*, or *E1c*. We refer to the *E1c* cells as wild-type (WT) control MEF cells, which, after tamoxifen-induced deletion of *Eset1*, are converted into *E1* (*Eset1*-null) MEFs. We used the CRISPR/Cas9 system in the *E1c* MEFs to introduce frameshift mutations within the catalytic domain of all the other SET-domain genes encoding H3K9 KMTs (Fig. 1a).

We first generated cell lines lacking the function of each set of paralogous genes. After transfecting *E1c* MEFs with plasmids carrying Cas9 and the respective guide RNAs, we isolated clones and confirmed the absence of detectable target proteins as well as the efficiency of tamoxifen-induced *Eset1* deletion by western blotting (Fig. 1b). We then assessed the contribution of each enzyme pair to the total levels of H3K9 methylation, by probing chromatin extracts with antibodies specific for H3K9me1, H3K9me2, and H3K9me3 (Fig. 1c). *Eset1/Eset2 (E1E2)* double-mutant MEFs do not display a significant reduction in overall H3K9 methylation levels. By contrast, *G9a/Glp (G9Gl)*-deficient cells have greatly lowered H3K9me1 and H3K9me2, while *Suv39h1/Suv39h2 (S1S2)* mutants lack most H3K9me3, and to a lesser extent, H3K9me2. Thus, the different H3K9 enzyme pairs have distinct functions in establishing H3K9 methylation, in agreement with published observations^5,8^.

### *Eset*-mutant MEF cells display micronuclei but maintain heterochromatic clustering

We visualized pericentric heterochromatin organization in these mutant MEF cells by performing DNA fluorescence in situ hybridization (DNA FISH) with probes specific for major satellite repeats (MSR). In WT (*E1c*) control cells, MSR sequences are clustered in bright and regular foci overlapping DAPI-dense areas (Fig. 1d). This organization is maintained in *Eset1*/*Eset2* or *G9a/Glp* double-deficient cells, and only *Suv39h1/Suv39h2* mutant cells display very modest aberrations in the definition of heterochromatic foci.

We then determined the integrity of the nuclear envelope by immunostaining with an antibody directed against the nuclear lamina component Lamin B. We did not observe any alteration in the morphology of the nuclear lamina of *G9a/Glp* and *Suv39h1/Suv39h2* mutant cells when compared to WT. In contrast, *Eset1/Eset2*-deficient cells have multiple micronuclei and display deformation of the nuclear lamina/envelope (Fig. 1e), suggesting that they harbor significant DNA damage.

These data indicate distinct functions for the Suv39h1/Suv39h2, Eset1/Eset2, and G9a/Glp systems in establishing H3K9 methylation states, as well as the different requirements for maintaining nuclear envelope morphology. However, none of these gene pair mutants show significant alterations in heterochromatin organization, suggesting a higher degree of redundancy between these three H3K9-methylating systems.

### Compound KMT-mutant MEF cells that lack the six SET-domain H3K9-methylating enzymes have lost all H3K9 methylation states

**Fig. 2:**
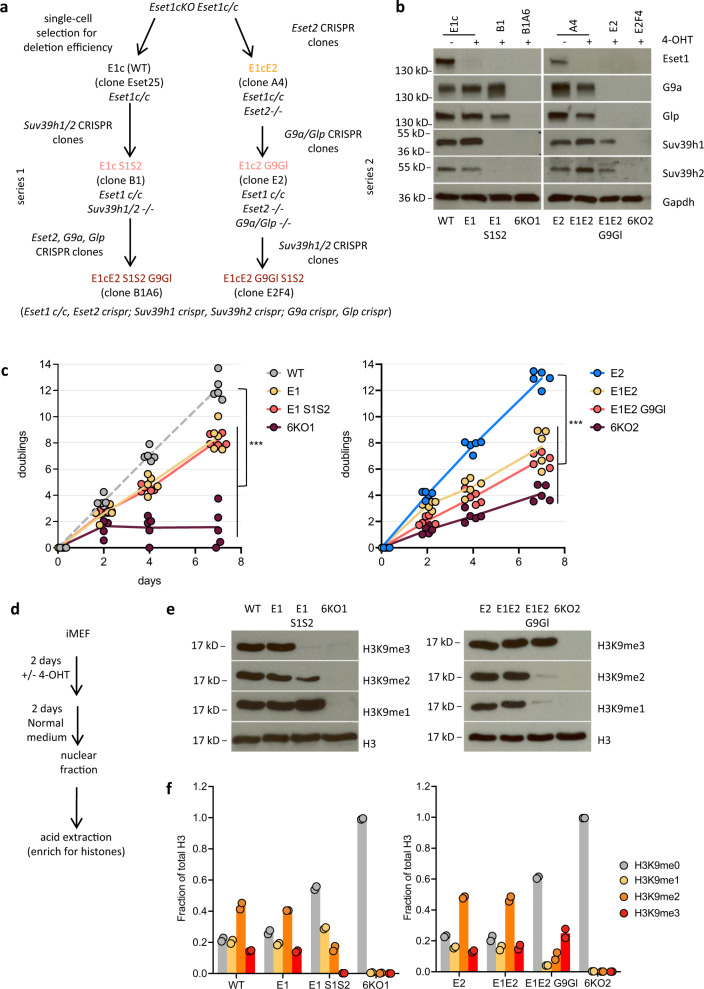
Loss of H3K9 methylation in compound H3K9 KMT-mutant MEF cells. **a** Generation of compound H3K9 KMT-mutant MEF by successive CRISPR/Cas9-mediated inactivation of genes encoding H3K9 KMT enzymes. Two series of H3K9 KMT mutants were generated. Nomenclature for the H3K9 KMT mutants is indicated on the scheme. *E1c* (*Eset1-*conditional, also WT), *E1* (*Eset1*-null), *E2* (*Eset2*-null), *G9* (*G9a*-null), *Gl* (*Glp*-null,) *S1* (*Suv39h1*-null), and *S2* (*Suv39h2*-null). **b** Western blot analysis for H3K9 KMT gene products in whole-cell extracts of WT and H3K9 KMT mutants. Gapdh was used as a loading control. Representative results of two independent experiments. Source data are provided as a Source Data file. **c** Proliferation capacity of compound H3K9 KMT mutants. The *y* axis indicates cumulative doublings after consecutive days in culture (*x* axis). *E1c*/WT and *E2* were grown in a normal medium, and all other H3K9 KMT mutants in presence of 1 μM 4-OHT in a culture medium (individual data points with line connecting their mean, *N* = 5 independent experiments). Statistically significant differences in doubling time, when compared to WT (left) or E2 (right) growth curves are shown by asterisks (***P = 1.1 × 10^−10^ for *E1* and *E1 S1S2*, *P* = 7.2 × 10^−11^ for *6KO1*, *P* = 2.8 × 10^−8^ for *E1E2, P* = 2.6 × 10^−8^ for *E1E2 G9Gl* and *6KO2*, extra-sum-of-squares *F* test). **d** Induction of *Eset1* deletion. Cells were grown for 2 days in presence of 1 μM 4-OHT in culture medium, followed by 2 days in normal medium without 4-OHT. After cell lysis and fractionation, histones were acid-extracted from the nuclear fraction. **e** Western blot analysis of H3K9 methylation states in compound H3K9 KMT mutants. Acid-extracted histones were probed with antibodies specific for H3K9me1, H3K9me2, and H3K9me3. Total H3 was used as a loading control. Representative results of three independent experiments. Source data are provided as a Source Data file. **f** Mass spectrometry analysis of H3K9 methylation states. The *y* axis indicates the fraction of total H3 carrying H3K9me1, H3K9me2, or H3K9me3 modifications, determined by normalizing intensities obtained for the corresponding peptides in nanoLC–MS (bars represent mean with individual data points overlaid, *N* = two independent samples).

To address this question, we next generated mutant cell lines with compound inactivation of multiple H3K9 SET-domain genes, in combination with the *Eset1*-conditional mutation. We proceeded stepwise to establish two series of mutant cell lines that progressively lack increasing numbers of H3K9-methylating enzymes (Fig. 2a). In the first series, we generated *Eset1*-conditional MEF cells lacking *Suv39h1* and *Suv39h2* (*E1c S1S2)* and further inactivated the remaining three H3K9 SET-domain genes to generate cells carrying mutations in all six H3K9-methylating enzymes (*E1cE2 S1S2 G9Gl*). In a parallel series, we first targeted *Eset2* (*E1cE2*), then *G9a* and *Glp* (*E1cE2 G9Gl*), and finally *Suv39h1* and *Suv39h2* (*E1cE2 G9Gl S1S2*). We confirmed the absence of mutant gene products, as well as the efficiency of tamoxifen-induced *Eset1* deletion by western blotting (Fig. 2b).

We first characterized the growth properties of these compound H3K9 KMT-mutant cell lines. While all compound H3K9 KMT cell lines are viable and proliferating when growing in the absence of tamoxifen (i.e., when *Eset1* is still functional), inducing *Eset1* deletion in the continuous presence of tamoxifen leads to cell death after a few days (i.e., more than 4 days) in culture (Fig. 2c). This lethality is more pronounced in cells lacking all six H3K9 KMT (*E1E2 S1S2 G9Gl* or abbreviated as *6KO1* and *E1E2 G9Gl S1S2* or abbreviated as *6KO2*) when compared to the single inactivation of *Eset1* (*E1*).

For the following experiments and sample preparations, we, therefore, induced *Eset1* deletion by a 2-day incubation with tamoxifen, followed by a 2-day culture in the normal medium (Fig. 2d).

We then examined H3K9 methylation in chromatin extracts from the various H3K9 KMT mutants by western blotting (Fig. 2e). Consistent with our previous observations with H3K9 SET-domain gene pair mutants, *Eset1* (*E1*), *Eset2* (*E2*), and *Eset1/Eset2* (*E1E2*)-deficient cells display comparable levels of H3K9 methylation as WT cells. Compound *Eset1* and *Suv39h1/Suv39h2* mutants (*E1 S1S2*) largely lack H3K9me3, while compound *Eset1*/*Eset2* and *G9a*/*Glp* mutants (*E1E2 G9Gl*) have drastically reduced H3K9me1 and H3K9me2. Strikingly, in extracts from the compound *6KO1* and *6KO2* mutants, we could not detect any residual H3K9me1, H3K9me2, or H3K9me3.

To validate this observation with an antibody-independent approach, we analyzed the same extracts by mass spectrometry (Fig. 2f). We detected very similar changes in H3K9 methylation states, and confirmed the nearly total absence of H3K9 methylation in chromatin extracted from the *6KO1* and *6KO2* mutants, with H3K9me1, H3K9me2, and H3K9me3 levels reduced to ≤1% as compared to WT.

We also investigated possible changes in the abundance of other histone modifications by mass spectrometry, and observed only a moderate increase in H3K9 acetylation or in H3K27me3, together with a decrease in H3S10 phosphorylation, specifically in extracts from the *6KO* mutants (Supplementary Fig. 1A). We also examined changes in total levels of H3 or H4 acetylation, as well as H3K4me3 by western blotting. While H3 acetylation was increased in some of the H3K9 KMT mutants, particularly in extracts from *6KO* cells, H4 acetylation and H3K4me3 were not significantly altered (Supplementary Fig. 1B). Therefore, loss of H3K9 methylation does not lead to a general or bulk increase in histone modifications associated with active chromatin, although the absence of H3K9 methylation might facilitate H3 acetylation. Importantly, these changes may not occur specifically at heterochromatin regions.

The presence of micronuclei in some of the H3K9 KMT mutants prompted us to examine the phosphorylation of the histone variant H2A.X (γH2A.X), a known marker of DNA double-strand breaks^17^. We observed an increase in γH2A.X signal in some of the H3K9 KMT mutants, particularly in extracts from *6KO1* cells (Supplementary Fig. 1C), but these levels remained low when compared to control cells treated with the topoisomerase inhibitor Etoposide and they were not apparent in extracts from *6KO2* cells. This might reflect different kinetics of DNA repair at heterochromatin regions and/or a somewhat altered involvement of heterochromatin proteins in the activation of DNA repair pathways^18^ between the *6KO1* and *6KO2* mutants. In addition, it has been shown that micronuclei can also form without γH2A.X accumulation^19^.

We further assessed global DNA methylation levels in the *6KO* mutant cells, either by DNA dot-blot analysis using an antibody recognizing 5-methylcytosine (5mC) (Supplementary Fig. 1D) or by mass spectrometry (Supplementary Fig. 1E). As controls, we used mouse ES cells that were either wild type or mutant for the three DNA methyltransferases establishing 5mC (*Dnmt* TKO)^20^. *Dnmt* TKO ES cells have only residual levels of 5mC and of 5-hydroxymethylcytosine (5hmC). In contrast, *6KO* MEF cells maintain high, although modestly decreased 5mC and 5hmC levels, corresponding to ~80% 5mC (*P* < 0.05) or 60% 5hmC (non-significant) of the signals observed in WT MEFs. These data indicate that loss of H3K9 methylation does not lead to an immediate and overall or genome-wide loss of DNA methylation.

### Massive derepression of repeat elements and retrotransposons in compound H3K9 KMT-mutant MEF cells

**Fig. 3:**
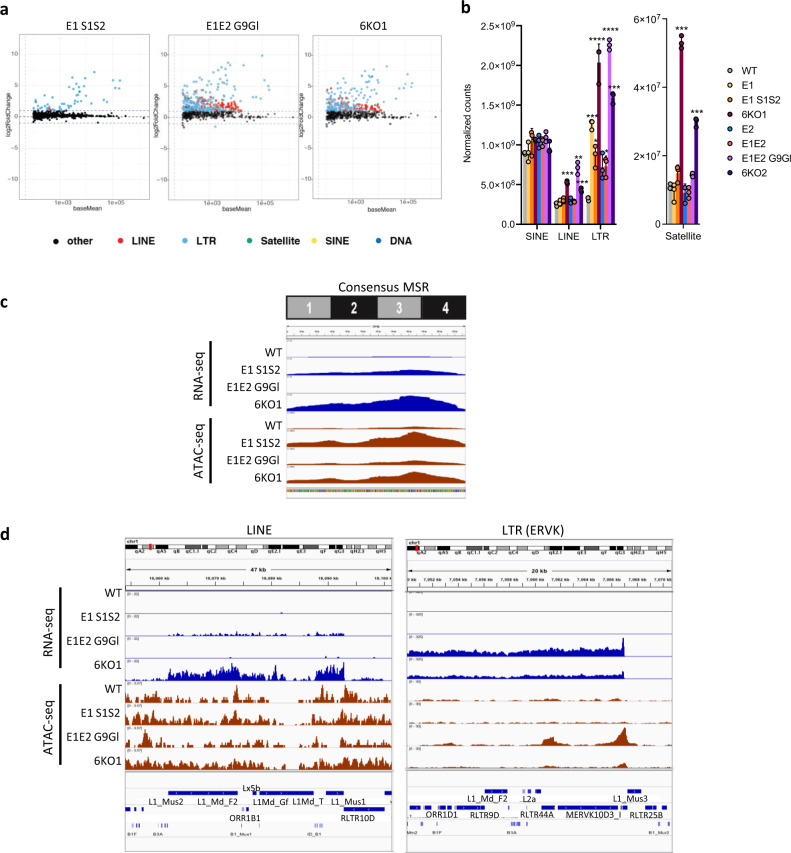
Massive derepression of repeat elements in compound H3K9 KMT-mutant MEF cells. **a** MA plots depicting changes in repeat element expression in compound H3K9 KMT mutants relative to WT, as determined by RNA sequencing (RNA-seq). From left to right: *E1 S1S2, E1E2 G9Gl*, and *6KO1*. Black dots represent repeat elements whose change in expression is not statistically significant. Red, LINE elements. Light blue, LTR retrotransposons. Green, Satellites. Yellow, SINE elements. Dark blue, DNA transposons. **b** Global changes in expression for distinct repeat classes (SINE, LINE, LTR, and Satellite), in each of the compound H3K9 KMT mutants. The *y* axis indicates normalized read counts (mean ± SD, individual data points overlaid, *N* = 3 independent samples). Satellites are shown on a separate graph due to their lower expression level. Asterisks indicate statistically significant differences when compared to WT levels (LINE: ***P = 1.1 × 10^−4^ for *6KO1*, **P = 0.006 for *E1E2 G9Gl*, ***P = 8.0 × 10^−4^ for *6KO2*; LTR: ***P = 3.7 × 10^−4^ for *E1*, *P = 0.019 for *E1 S1S2*, ****P = 6 × 10^−5^ for *6KO1*, *P = 0.026 for *E1E2*, ****P = 8.4 × 10^−5^ for *E1E2 G9Gl*, ***P = 3.6 ×10^−4^ for *6KO2*; Satellite, ***P = 1.2 × 10^−4^ for *6KO1* and *P* = 1.4 × 10^−4^ for *6KO2*, two-sided, unpaired *t* test). **c** Coverage plot of major satellite repeat (MSR) transcripts to the consensus MSR sequence in WT and H3K9 KMT mutants (from top to bottom: *E1 S1S2*, *E1E2 G9Gl*, and *6KO1*). ATAC-seq indicating DNA accessibility in the same mutants is shown below. **d** Genome browser tracks showing expression levels (top) and ATAC-seq profiles (bottom) of example genomic regions with repeat elements. Left, genomic region harboring LINE elements. Right, region with LTR-containing elements (ERVK).

We determined the transcriptional response of H3K9 KMT disruption by performing RNA sequencing (RNA-seq) in the various H3K9 KMT-mutant cell lines. We first focused on all repetitive elements in the mouse genome. As previously reported^6,21^, deletion of *Eset1* alone is sufficient to induce derepression of a subset of endogenous retroviruses (ERV) (Fig. 3b and Supplementary Fig. 2). Notably, the numbers of derepressed ERV elements, as well as the magnitude of their upregulation, are increased in cell lines where additionally also *G9a* and *Glp* are deleted (*E1E2 G9Gl*) and further augmented in the *6KO1* and *6KO2* cells (Fig. 3a, b). Among the distinct ERV subtypes, ERV class I elements of the ERV1 family are most sensitive to *Eset1* depletion (Supplementary Fig. 2A). ERV class II elements, such as the ERVK family, show only a modest increase in expression in cells lacking *Eset1*, or both *Eset1* and *Eset2* (*E1E2*), but are strongly upregulated in *E1E2 G9aGl* mutants and in *6KO1* and *6KO2* cells (Fig. 3c). In contrast, expression of ERV class III elements is either unaltered (ERVL), or only moderately increased (MaLR), even in mutant cells lacking all six KMT (Supplementary Fig. 2A).

Non-long terminal repeat (LTR) containing retrotransposons are classified as short or long interspersed nuclear elements (SINE or LINE). While expressed at relatively high levels in WT cells, SINE is not dysregulated by the lack of H3K9 KMT (Fig. 3b). LINE elements, in particular LINE L1, are overexpressed specifically in H3K9 KMT-mutant cells lacking *G9a* and *Glp* (*E1E2 G9Gl*) and in *6KO1* and *6KO2* cells (Fig. 3a, b).

Satellite DNA forms large tandem repeat arrays at centromeric and pericentromeric regions, but can also be found in a few intergenic regions. In particular, major satellite repeats (MSR) are repressed by Suv39h enzymes in various cell types, including fibroblasts^22^. Satellite repeats are exclusively derepressed in mutant cells lacking Suv39h enzymes, and not in other compound H3K9 KMT mutants (Fig. 3b). In cells depleted for *Suv39h1/Suv39h2* together with *Eset1*, the only upregulated satellite repeats are MSR (annotated as GSAT_MM in Supplementary Fig. 2A). Interestingly, MSR expression is further increased in cells that lack all 6 KMT, indicating that while Suv39h are the chief H3K9-methylating enzymes for MSR repression, other H3K9 KMT can participate in their control (Fig. 3c and Supplementary Fig. 2A). In addition to MSR, *6KO1* and *6KO2* mutant cells also upregulate MMSAT4 sequences (Supplementary Fig. 2A).

We also assessed changes in chromatin accessibility by ATAC-seq. The disruption of H3K9 KMT leads to a global increase in chromatin accessibility over repeat elements, which is particularly pronounced for MSR sequences (Fig. 3c, d and Supplementary Fig. 2B). Surprisingly, however, changes in chromatin accessibility did not strictly reflect changes in repeat expression, and several repeat types, such as ERVL, gained chromatin accessibility without being derepressed (Supplementary Fig. 2B).

In summary, the disruption of H3K9 KMT leads to the derepression of multiple families of repeat elements. These results also reveal that distinct H3K9 KMT have both specific and overlapping functions in the silencing of repeat elements.

### Activation of pattern-recognition and DNA damage pathways in compound H3K9 KMT-mutant MEF cells

**Fig. 4:**
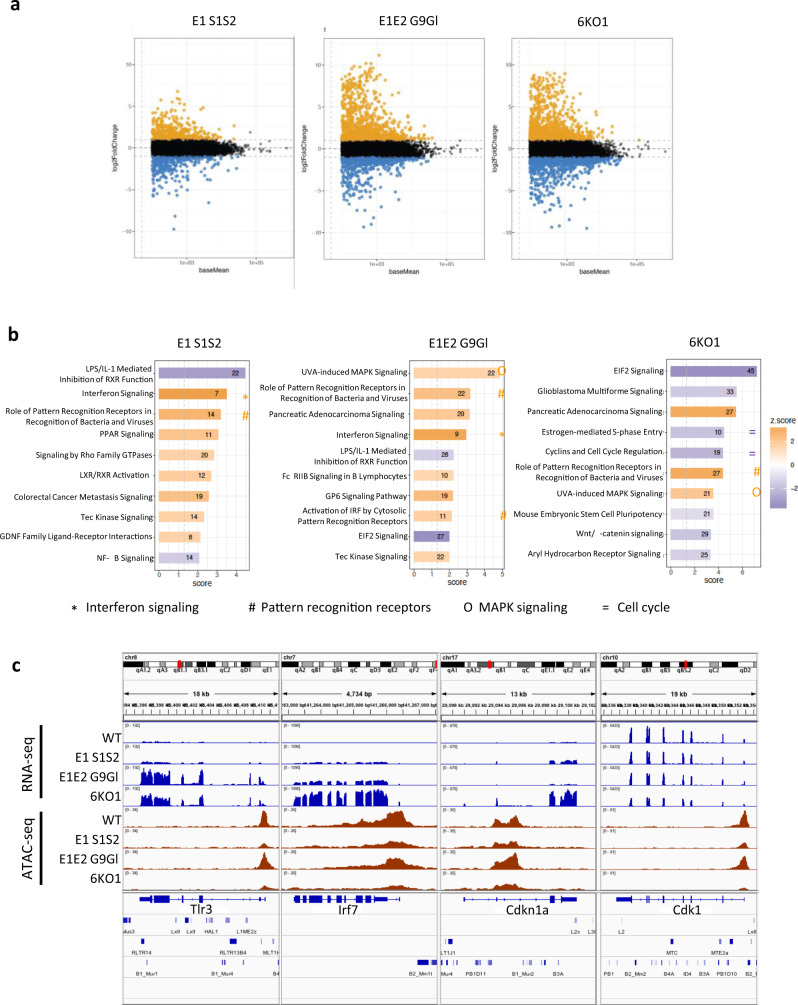
Changes in gene expression in compound H3K9 KMT-mutant MEF cells. **a** MA plots depicting changes in gene expression in compound H3K9 KMT mutants relative to WT, as determined by RNA sequencing (RNA-seq). From left to right: *E1 S1S2, E1E2 G9Gl*, and *6KO1*. Black dots represent genes whose changes in expression are not statistically significant. Yellow dots represent upregulated genes, and blue dots downregulated genes. **b** Ingenuity pathway analysis (IPA) of genes differentially expressed in compound H3K9 KMT mutants relative to WT. From left to right: *E1 S1S2, E1E2 G9Gl*, and *6KO1*. The *x* axis indicates fold change, and the color code indicate the *z* score for each category (legend on the right). Orange indicates upregulation, and purple downregulation of the pathway. Pathways related to pattern-recognition receptors, interferon signaling, MAP kinase signaling, or cell cycle regulation are highlighted by symbols as indicated below the table. **c** Genome browser tracks showing RNA expression levels (top) or ATAC accessibility (bottom) of example genes in compound H3K9 KMT mutants. From left to right, Toll-like receptor 3 (*Tlr3*), interferon regulatory factor 7 (*Irf7*), cyclin-dependent kinase inhibitor 1 A (*Cdkn1a*), and cyclin-dependent kinase 1 (*Cdk1*). For both RNA-seq and ATAC-seq tracks, the top profile corresponds to WT, followed from top to bottom by *E1 S1S2, E1E2 G9Gl*, and *6KO1* mutants.

We next analyzed changes in gene expression levels upon loss of H3K9 methylation. We observed dysregulation of increasing numbers of genes in H3K9 KMT mutants lacking progressively more H3K9 KMT (Fig. 4a). While *Eset1* mutant cells display 362 upregulated and 331 downregulated genes (fold change ≥2, *P* value ≤ 0.01), when compared to WT control cells, cells depleted for all six KMT show deregulation of thousands of genes (3427 up and 1632 down in *6KO1* and 2517 up and 897 down in *6KO2*).

We determined the functional classification of these deregulated genes with the Ingenuity Pathway Analysis database (Fig. 4b). Among the most significantly enriched pathways, pattern-recognition receptors were consistently upregulated. This included, among other genes, the *Tlr3* and *Tlr4* genes, encoding Toll-like receptors, as well as *Ddx58*/*Rig-I* (Fig. 4c). This is consistent with previous studies where treatment of cancer cells with DNMT inhibitors led to overexpression of ERV, which triggered the activation of the double-stranded RNA sensor TLR3 and, in turn, a type I interferon response^23,24^. Accordingly, H3K9 KMT-deficient MEF cells also display activation of interferon signaling (significantly enriched pathway in all H3K9 KMT-mutant cells except *Eset2* mutants), including overexpression of type I interferon regulators *Irf7, Irf9*, and *Stat1* (Fig. 4c).

Other significantly altered pathways indicate activation of DNA damage-sensing mechanisms. This includes MAP kinase signaling, with upregulation of *Mapk10, Mapk11*, and *Pik3r5*, among others, and of the p53 pathway, with increased expression of *Trp53inp1*, *Rb1*, and *Cdkn1a* (Fig. 4c). In agreement with the growth defect exhibited by the H3K9 KMT-mutant cell lines, we also observed reduced expression of multiple cyclin-dependent kinases (*Cdk1, Cdk2, Cdk4, Cdk6*, and *Cdk7*, Fig. 4c), and upregulation of apoptosis-related factors, such as *Casp7* and multiple poly (ADP-ribose) polymerases.

Collectively, these results indicate that loss of H3K9 KMT induces a signaling cascade, including DNA damage-sensing pathways, interferon response, attenuation of cell proliferation, and cell death. The activation of pattern-recognition receptors, such as *Tlr3* and *Rig-I*, suggests that a major cause of these defects could be the massive derepression of repeat elements, primarily ERV, that we observed in the H3K9 KMT-mutant cells.

### Dysregulation of H3K9me-positive vs. H3K9me-negative genes in H3K9 KMT-mutant MEF cells

We wondered to what extent these changes in gene expression would reflect direct participation of H3K9 methylation. Since comprehensive datasets for all three H3K9 methylation states are not available in mouse fibroblasts, we generated reference maps for H3K9me1, H3K9me2, and H3K9me3 in WT (*E1c*) MEFs. We first validated the antibodies by ChIP-qPCR at known target loci (Supplementary Fig. 3A) and then performed chromatin immunoprecipitation followed by deep sequencing (ChIP-seq). Peak calling identified 42893 regions of enrichment for H3K9me1, 29285 for H3K9me2, and 27281 for H3K9me3. Distinct H3K9 methylation states were often observed in overlapping genomic intervals. However, H3K9me1 peaks had only low signals for H3K9me2 and H3K9me3, H3K9me2 peaks coincided with broad signals for H3K9me3 and H3K9me3 peaks also had some enrichment for H3K9me2 (Supplementary Fig. 3B, top panels). In total, 559 Mb, corresponding to 21% of the mouse genome were decorated by H3K9 methylation in WT MEFs.

A large fraction of H3K9 methylated regions corresponded to intergenic and intronic sequences, ranging from 55% for H3K9me1 to 69% for H3K9me2 and 70% for H3K9me3 (Supplementary Fig. 3C). A smaller subset of regions overlapped with gene promoters, defined as sequences within + /− 1 kilobase (kb) of the transcription start site (TSS). This was the case for 19% of H3K9me1 and around 10% for H3K9me2 or H3K9me3-enriched regions. We identified 4289 genes that are decorated by H3K9me1 at their promoters (e.g., *Tcap* and *Cacna1i*), 1567 genes with H3K9me2-positive promoters (e.g., *Defb40* and *Olfr1431*), and 1415 genes with promoters enriched for H3K9me3 (e.g., *Nkx6-1* and *Bmp2*) (see Supplementary Fig. 4A). The majority of H3K9me1-positive promoters (3179 genes) were devoid of H3K9me2 and H3K9me3. By contrast, H3K9me2 and H3K9me3 often decorated the same gene promoters and only 270 or 462 genes were predominantly marked by H3K9me2 or H3K9me3 (Supplementary Fig. 4B). In total, we detected H3K9 methylation at the promoters of 5297 genes.

**Fig. 5:**
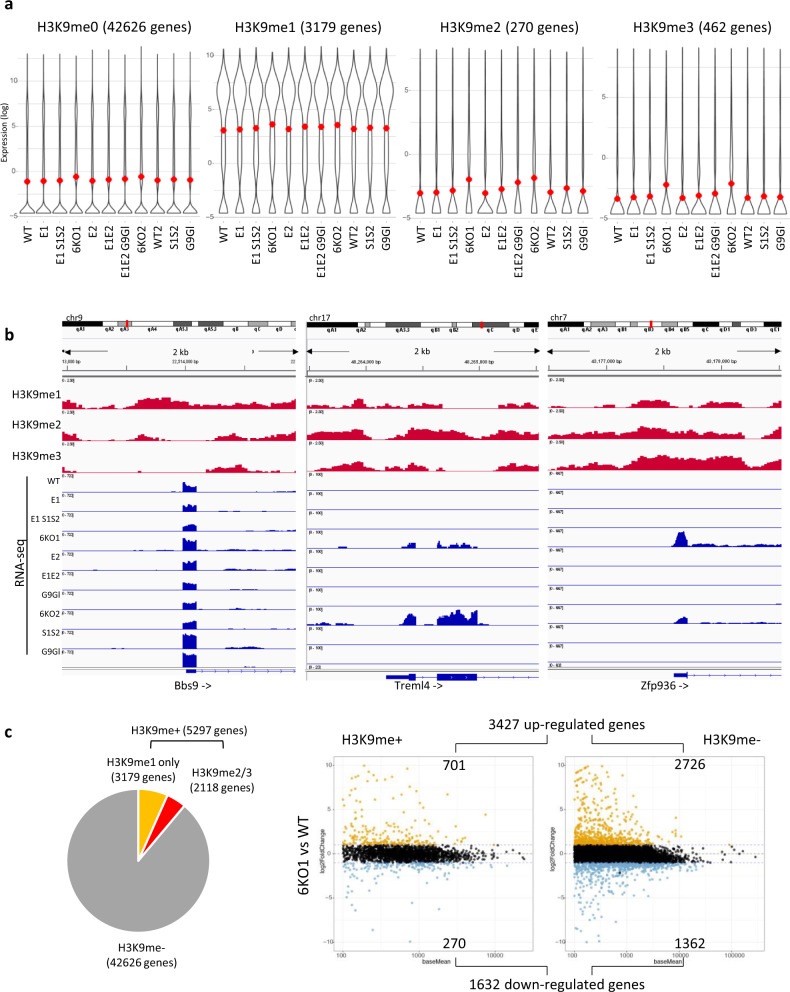
Dysregulation of H3K9me-positive vs H3K9me-negative genes in compound H3K9 KMT-mutant MEF cells. **a** Differential expression of genes with H3K9 methylated promoter regions. Violin plots representing the distribution of gene expression levels in WT and compound H3K9 KMT mutants, as determined by RNA sequencing (RNA-seq, log scale). Red dots indicate mean expression level. The genes are grouped according to H3K9 methylation status over their promoter region, as determined by chromatin immunoprecipitation sequencing (ChIP-seq) in WT MEFs. Promoters are defined as within + /−1 kilobase (kb) from the transcription start site (TSS). From left to right, genes with promoters lacking any H3K9 methylation peak (H3K9me0, 42626 genes), followed by genes with promoters positive for H3K9me1 (3179 genes), H3K9me2 (270 genes), or H3K9me3 (462 genes). It is important to note that this analysis does not include genes with promoter regions that show enrichment for multiple H3K9 methylation states (as summarized in Supplementary Fig. 4B). **b** Genome browser tracks showing H3K9me1, H3K9me2, or H3K9me3 enrichment (log2 fold change, red) over example gene promoters, as determined by ChIP-seq in WT MEFs. From left to right, Bardet–Biedl syndrome 9 (*Bbs9*, H3K9me1-positive), Triggering receptor expressed on myeloid cells 4 (*Treml4*, H3K9me2-positive) and Zinc finger protein 936 (*Zfp936*, H3K9me3-positive). Bottom shows RNA expression (RNA-seq, blue) in WT and compound H3K9 KMT mutants. Each browser track represents a 2 kb genomic interval surrounding the TSS. **c** Pie chart of genes classified by H3K9 methylation status at their promoter regions (left). Right, MA plots depicting changes in gene expression (RNA-seq) in *6KO1* cells relative to WT, shown for genes with (H3K9me + ) or without (H3K9me-) H3K9 promoter methylation. Black dots represent genes whose changes in expression are not statistically significant. Yellow dots represent upregulated genes and blue dots downregulated genes. The total number of genes upregulated in *6KO1* cells is indicated above the plots, and the number of genes downregulated is shown below. The numbers of H3K9me+ and H3K9me- up- or downregulated genes are indicated on the corresponding sector of the MA plots. For graphical representation, only genes with base mean >100 are shown on the plots.

We then used our RNA-seq data to determine the expression status of these H3K9me-positive genes in WT (*E1c*) MEFs and in the various H3K9 KMT-mutant cell lines. For this, we applied a meta-analysis that illustrates gene expression in violin plots. Surprisingly, genes with H3K9me1-marked promoters have significantly (*P* < 2.2 × 10^−16^) higher expression levels as compared to genes without H3K9 methylation (H3K9me0 genes in the left panel of Fig. 5a), and this elevated expression was largely unaltered in all of the H3K9 KMT mutants (Fig. 5a and browser shot for example gene *Bbs9* in Fig. 5b). By contrast and as expected, genes with H3K9me2- or H3K9me3-marked promoters were silenced and had significantly (*P* < 2.2 × 10^−16^) reduced or no expression levels as compared to H3K9me-negative (H3K9me0) genes (Fig. 5a). The H3K9me2 mediated gene silencing was weakened (*P* < 0.01) in *E1E2 G9Gl* compound H3K9 KMT-mutant cells (Fig. 5a). Both H3K9me2 and H3K9me3 mediated gene silencing was partly lost (*P* < 0.001) in 6KO1 and 6KO2 cells (Fig. 5a and browser shots for example genes *Treml4* and *Zfp936* in Fig. 5b). However, it is important to note that loss of H3K9 methylation only caused desilencing in a minor fraction of the H3K9me2- or H3K9me3-positive genes: with a twofold cutoff (and a base mean of >100 reads), 226 of 270 H3K9me2-positive genes, and 390 of 462 H3K9me3-positive genes were not significantly upregulated in any of the H3K9 KMT mutants, not even in the *6KO1* or *6KO2* cells.

**Fig. 6:**
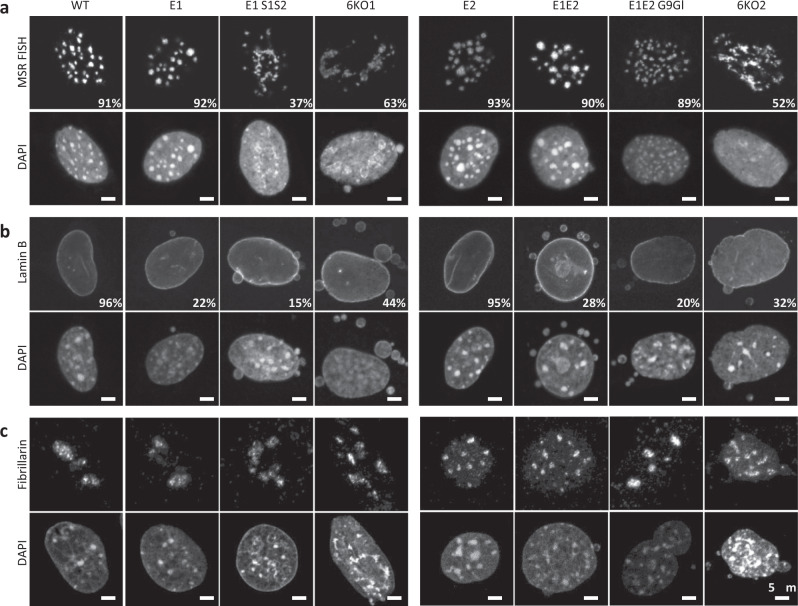
Collapse of heterochromatin in compound H3K9 KMT-mutant MEF cells. **a** DNA FISH for major satellite repeats (MSR) in control and compound H3K9 KMT mutant. Percentages indicate cells that display the shown pattern. *N* = 501 cells examined for WT, 303 for *E1*, 352 for *E1 S1S2*, 304 for *6KO1*, 308 for *E2*, 311 for *E1E2*, 301 for *E1E2 G9Gl*, and 309 for *6KO2* over three independent experiments. For each image, lower panels show DAPI counterstaining of the same nuclei. **b** Immunofluorescence for Lamin B, marking the nuclear lamina. Percentages indicate cells that display the shown pattern. *N* = 680 cells examined for WT, 785 for *E1*, 302 for *E1 S1S2*, 305 for *6KO1*, 573 for *E2*, 499 for *E1E2*, 558 for *E1E2 G9Gl,* and 427 for *6KO2* over three independent experiments. For each image, lower panels show DAPI counterstaining of the same nuclei. **c** Immunofluorescence for Fibrillarin, marking nucleoli. Representative images (*N* = 100 cells) are shown. For each image, lower panels show DAPI counterstaining of the same nuclei. Scale bars represent 5 μm.

We further focused on the *6KO* mutants and analyzed changes in gene expression (as compared to WT) separately for H3K9me-positive and for H3K9me-negative genes (H3K9me+ and H3K9me- genes, Fig. 5c). While H3K9me-positive genes represent 11% (5297 genes) of all genes, they account for >20% of the upregulated genes in the *6KO1* cells (701 of 3427 genes) (Fig. 5c) or *6KO2* cells (508 of 2517 genes), indicating an increased response of H3K9me-positive genes to loss of H3K9 methylation. By contrast, 80% of the genes derepressed in the *6KO1* mutants (2726 of 3427 genes) do not have H3K9 methylation at their promoters in WT cells. While some of these H3K9me-negative genes might still be regulated by H3K9 methylation at distal sites, such as enhancers, this result suggests that the majority of the genes dysregulated in the compound H3K9 KMT mutants are not direct targets for the H3K9 KMT systems. Instead, we propose that the failure of repeat repression and the induction of DNA damage and stress signaling pathways in the compound H3K9 KMT mutants leads to widespread changes in gene expression.

### Partial dispersion of heterochromatic foci in *Eset*/*Suv39h* mutant MEF cells

We visualized heterochromatin organization in the series of compound H3K9 KMT-mutant MEF cells by FISH for MSR sequences (Fig. 6a). As described above (Fig. 1), *Eset1*, *Eset2*, or *Eset1/Eset2* double-mutant cells display a nuclear clustering of MSR sequences that is indistinguishable from WT cells. MSR clustering is also maintained in *E1E2 G9Gl* deficient cells, although they display an increased number of smaller heterochromatin foci. By contrast, cells with a compound deficiency of Eset1 and Suv39h1/Suv39h2 enzymes (*E1 S1S2*) show a partial dispersion of heterochromatin, with a significant fraction of the cells (37%) displaying elongated or generally irregular MSR foci (Fig. 6a). We also stained these cells with antibodies for H3K9me3 and the heterochromatin protein HP1α (Supplementary Fig. 5A, B). Both H3K9me3 and HP1α are completely dispersed in the nuclei of the *E1 S1S2* mutant cells. This is not detected in compound H3K9 KMT mutants with functional Suv39h enzymes, even when all of the other four H3K9 KMT are inactivated (*E1E2 G9Gl*). We verified the specificity of these observations by analyzing the nuclear distribution of Hmga1, a protein that binds AT-rich sequences within pericentric chromatin^25,26^. In all compound H3K9 KMT mutants, Hmga1 signals remain intact and overlap with the DAPI-dense regions (Supplementary Fig. 5C).

To further investigate nuclear organization in the H3K9 KMT mutants, we visualized the integrity of the nuclear lamina by immunofluorescence detection of Lamin B (Fig. 6b). As we observed for *Eset1/Eset2* double-null cells, all compound H3K9 KMT mutants lacking *Eset1* function displayed multiple micronuclei and deformation of the nuclear lamina/envelope, in agreement with these cells expressing markers of DNA damage (see Supplementary Fig. 1C). We also analyzed the distribution of Fibrillarin, a protein-labeling nucleoli, and did not observe significant differences between the various compound H3K9 KMT mutants (Fig. 6c). These data suggest that the global organization of subnuclear compartments is not drastically altered in the H3K9 KMT mutants. Together, these observations confirmed that the spatial organization of heterochromatin is mostly dependent on Suv39h enzymes, although an additional inactivation of Eset (and probable defects in the nuclear lamina) in a Suv39h-deficient background is required for a partial breakdown of heterochromatin clustering, as previously reported^13^.

### Full collapse of heterochromatin in 6KO H3K9 KMT MEF cells

We next analyzed heterochromatin organization in MEF cells lacking all 6 KMT. Strikingly, FISH staining for MSR sequences revealed a complete collapse of heterochromatin foci in both *6KO1* and *6KO2* mutants (Fig. 6a). While not fully penetrant, this phenotype was observed in the majority of nuclei of both *6KO1* (63%) and *6KO2* (52%) cells. As for the *E1 S1S2* cells, H3K9me3 and HP1α were also completely dispersed (Supplementary Fig. 5A, B). Hmga1 remained enriched over DAPI-bright regions, even though these DAPI signals displayed an aberrant and non-clustered organization (Supplementary Fig. 5C). Since Hmga1 was shown to also contribute to the integrity of heterochromatin foci^26^, its retention at the A/T-rich MSR-repeat sequences might explain, at least in part, the incomplete penetrance of heterochromatin dispersion in the *6KO* MEF cells.

Staining for Lamin B recapitulated defects in the nuclear lamina/envelope, as observed in all H3K9 KMT mutants lacking *Eset1* function, although the proportion of cells with micronuclei and the size of the micronuclei appeared increased in *6KO1* and *6KO2* cells (Fig. 6b). Fibrillarin staining indicated that the nucleoli structure was not significantly altered (Fig. 6c). Thus, complete loss of H3K9 methylation breaks heterochromatin clustering and reveals a definite function for H3K9 methylation in maintaining heterochromatin organization and genome integrity.

### 6KO H3K9 KMT MEF cells have lost electron-dense heterochromatin

**Fig. 7:**
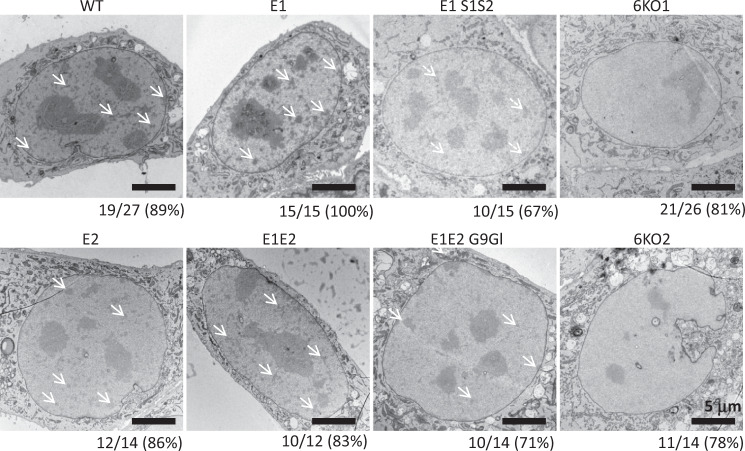
Loss of electron-dense heterochromatin in MEF cells lacking all 6 SET-domain H3K9 KMT. Transmission electron microscopy (TEM) images of nuclei from compound H3K9 KMT mutants. Each panel shows a representative TEM image of a nucleus of the indicated H3K9 KMT mutant. White arrows point to electron-dense heterochromatin clusters. The total number of cells analyzed and the percentage of cells displaying the shown pattern are indicated below each image. Scale bars represent 5 μm.

We analyzed this collapse of heterochromatin at higher resolution by performing transmission electron microscopy (TEM) in the various H3K9 KMT mutants (Fig. 7). In WT (*E1c*) cells, heterochromatin is readily visualized as small clusters of electron-dense material, often localized close to the nuclear envelope (Fig. 7, white arrows), while larger electron-dense regions correspond to nucleoli. *Eset1* (*E1*), *Eset2* (*E2*), and *Eset1/Eset2* (*E1E2*) double mutants display a similar distribution of electron-dense heterochromatin as compared to wild type, with >80% of cells displaying clearly defined heterochromatin clusters. Compound H3K9 KMT mutants lacking *Eset1* and *Suv39h*/*Suv39h2* (*E1 S1S2*) and, to some extent, *Eset1/Eset2*, *G9a/Glp* (*E1E2 G9Gl*) show reduced electron density or lower number of foci, but maintain the distinction between heterochromatin and euchromatin. Strikingly, this is not the case for *6KO1* and *6KO2* MEF cells: in half of these cells analyzed by TEM, no heterochromatin clusters could be detected, although nucleoli are preserved (Fig. 7) and a further fraction of the *6KO1* and *6KO2* cells displayed markedly reduced electron density and only very faint foci. Around 81% (*6KO1*, *n* = 26) or 78% (*6KO2*, *n* = 14) of the cells have lost the clear distinction of electron-dense heterochromatin. These data provide compelling evidence for the crucial requirement of H3K9 methylation in maintaining heterochromatin organization.

## Discussion

Heterochromatin forms distinct chromatin domains that can be visualized by DAPI staining of A/T-rich major satellite repeats in mouse cells. Suv39h enzymes are the central KMT for heterochromatic H3K9me3. However, while heterochromatin is weakened in *Suv39h* double-null cells, its overall organization and clustering remain largely intact. Many mechanisms could contribute to this heterochromatin stability independently of H3K9 methylation, including specific chromosome conformations, such as the Rabl configuration or anchoring to the nuclear membrane or other subnuclear structures^3,27,28^. For example, the three-dimensional organization of chromosomes in *S. pombe* involves both H3K9 methylation, which controls chromatin compaction near centromeres, and cohesin-mediated formation of globular domains along chromosome arms^27^.

Another possibility is that other silencing systems can partially compensate for the loss of Suv39h enzyme function, such as Polycomb Repressive Complexes (PRC) and/or DNA methylation. We indeed observed a moderate increase in H3K27me3 levels in *6KO* MEF cells chromatin, while global DNA methylation was only slightly decreased over the examined number of cell divisions. Recruitment of Polycomb-mediated H3K27me3 at heterochromatin has already been observed in other contexts where H3K9 methylation is reduced^5,29,30^.

The only minor decline of overall 5mC levels seems incoherent with the well-documented interdependence between H3K9 chromatin and CpG DNA methylation^31^. However, in lineage-committed cells, reduction of H3K9 methylation does not cause a global loss of DNA methylation at heterochromatin, as, for example, *Suv39h* double-null MEF cells keep high levels of 5mC at many repeat elements^32^. Maintenance of DNA methylation by Dnmt1 is indeed not strictly dependent on recruitment to H3K9 methylated nucleosomes^33,34^. Further, CpG methylation is not restricted to heterochromatin regions, but also abundant on transcribed gene bodies, where it is established by H3K36me3-mediated recruitment of Dnmt3b^35^. Determining whether and how these other silencing pathways are redirected or adjusted in the H3K9 KMT mutants would require genome-wide ChIP mapping of H3K27me3 and genome-wide bisulfite sequencing for 5mC.

We observed a high incidence of micronuclei and aberrant shape of the nuclear lamina/envelope in compound H3K9 KMT-mutant cells that correlated with transcriptional signatures indicating the activation of DNA damage pathways. This is in agreement with the known roles of heterochromatin in the maintenance of genome integrity that include protecting cells from replication stress, chromosome segregation errors, or aberrant recombination of repeats, as it was reviewed recently^3^. Interestingly, these defects were most pronounced in H3K9 KMT mutants where *Eset1* is inactivated and they also occurred in chromatin that maintains high levels of H3K9 methylation. These data suggest that in addition to the H3K9 histone, Eset1 has other non-histone substrates, some of which could function in the control of DNA damage and in correcting replication errors. Increased DNA damage was reported following *Eset1* deletion in mouse oocytes or in intestinal epithelium^36,37^.

The *6KO* H3K9 KMT display a collapse of heterochromatin foci and loss of electron-dense heterochromatin. This reveals an essential function for H3K9 methylation in maintaining heterochromatin as a separate nuclear domain. Our data also show a larger degree of cooperation among the 6 SET-domain H3K9 KMT to secure heterochromatin integrity. Conversely, this seems to exclude a contribution to H3K9 methylation and heterochromatin organization of other KMT systems, such as split SET-domain enzymes or “seven-beta-strand” (7βs) KMT^38^. The described dispersion of heterochromatin in *Prdm3*/*Prdm16* shRNA MEF mutants^13^ is probably related to concurrent secondary defects in the structuring of the nuclear lamina and not caused by a reduction of H3K9 methylation in nuclear chromatin.

H3K9 KMT mutants of any distinct gene pair of either *Suv39h1*/*Suv39h2*, *Eset1*/*Eset2*, or *G9a*/*Glp* maintain heterochromatin structure, indicating that functional redundancy between these SET-domain H3K9 KMT ensures the robustness of heterochromatin organization. Importantly, the contribution of each of these six SET-domain H3K9 KMT may be different in other cell types. For example, *Suv39h1*-null T cells start to disintegrate heterochromatic foci, even though the other 5 H3K9 KMT remain functional^39^. Similarly, *Eset1/Suv39h* compound mutant hepatocytes also have severely reduced EM-dense heterochromatin^40^.

What is the mechanism for the collapse of heterochromatin in the compound H3K9 KMT mutants? Chromatin that is deficient for all H3K9 methylation states will not provide binding for HP1 proteins and may have lost the physicochemical properties to engage in phase separation^3,41,42^. While the dispersion of HP1 binding is not sufficient to break heterochromatin^11^, other H3K9me2 or H3K9me1-binding proteins might substitute for HP1 in *Suv39h*-deficient cells, but not in compound H3K9 KMT-mutant cells that lack all H3K9 methylation states. Possibly, some of these putative H3K9-methyl binders could also assist in phase separation, and thus increase heterochromatin stability. Alternatively, there could also be non-histone substrates for the distinct SET-domain H3K9 KMT, particularly for Eset, that are needed for heterochromatin organization but fail to become methylated in the *6KO* MEF cells.

Several proteins were identified as candidate anchoring factors for heterochromatin. In mouse cells, Lamin B receptor (LBR) or Lamin A/C tether heterochromatin to the inner nuclear membrane in a cell-type-specific manner, but the interaction is likely indirect and adaptor proteins remain to be identified^43^. In *C. elegans*, the chromodomain containing protein CEC-4 binds methylated H3K9 and is required for recruitment of heterochromatin at the nuclear periphery, but dispensable for its silencing^44^. While no clear homolog of CEC-4 is found in mammals, other chromodomain proteins might function in a similar way.

Answering these questions will require future studies and would be facilitated if conditions for compound H3K9 KMT-mutant cells can be generated that allow the analysis of collapsed heterochromatin not only at its onset (this study) but also for more prolonged cell divisions. In summary, our work provides compelling evidence for a crucial function of H3K9 methylation in heterochromatin organization.

## Methods

### *Eset1*-conditional MEF

*Eset1*-conditional MEFs were described previously^6^. To optimize deletion efficiency, we sorted single cells in 96-well plates and selected for cell clones with high Cre activity (clone *Eset25*, referred to as *E1c*). After CRISPR/Cas9-mediated gene disruption (see below), we further re-introduced a construct expressing tamoxifen-inducible Cre together with a puromycin resistance gene (pCAGGS-CreERT2-EGFP-IRES-PURO), and maintained the cell lines under constant puromycin selection. Cre-mediated deletion of exons 15 and 16 of *Eset1* was induced by growing the cells in a medium containing 1 μM (Z)-4-hydroxytamoxifen (4-OHT, Sigma Aldrich, H7904) for 2 days, followed by 2 days in normal medium, to minimize potential side effects of prolonged growth in presence of tamoxifen. Deletion efficiency was assessed by RT-qPCR with primers specific for the deleted exons, or by western blot for the Eset1 protein. We refer to *E1c* as *WT*.

### CRISPR/Cas9 mutations

CRISPR/Cas9-mediated gene disruptions were performed by transfection of chimeric sgRNA/Cas9 expression vectors^45^. For each SET-domain KMT target gene, two unique guide RNAs were designed using online tools (CRISPR.mit.edu) and subsequently cloned in the chimeric sgRNA/Cas9 expression vectors pSpCas9(BB)-2A-Puro (pX459) or pSpCas9(BB) (pX330), both from Addgene. Cells were transfected with Xfect (Clontech) and selected for 2 days in puromycin-containing media. After selection, the efficiency of Cas9-mediated cleavage was evaluated by Surveyor assay^45^. Puromycin-resistant cells were further sorted to single cells in 96-well plates, and individual clones analyzed for the absence of functional protein by western blotting when specific antibodies were available, or for the absence of a functional copy of the gene by sequencing the relevant exons (for *Eset2*). The sequences of the guide RNAs used in this study are listed in Supplementary Table 1.

### Gene pair mutations

For gene pair mutants, we transfected combinations of chimeric sgRNA/Cas9 expression vectors in *E1c* MEFs and selected mutant clones as described above. Conditional *Eset1,* Eset2-null mutants (*E1cE2*) were obtained by introducing a single guide RNA specific for *Eset2* into *E1c* MEFs. Clone A4 was selected after sequencing revealed a frameshift mutation within exon 7 of *Eset2*. *G9a/Glp* double mutants were generated by co-transfection of two sgRNA/Cas9 vectors targeting *G9a* and *Glp*. Clone H7 was selected after western blot analysis confirmed the absence of G9a or Glp proteins. Suv39h-deficient cells were generated by co-transfection of two sgRNA/Cas9 vectors targeting *Suv39h1* and *Suv39h2* in *E1c* MEFs. Clone B1 was selected after western blot analysis confirmed the absence of Suv39h1 or Suv39h2 proteins.

### Compound SET-domain H3K9 KMT mutants

Compound H3K9 KMT mutants were generated by successive rounds of sgRNA/Cas9 transfection and selection of relevant clones. The nomenclature of the clones refers to null mutation of SET-domain KMT genes and is indicated as *E1* (*Eset1*-null), *E2* (*Eset2*-null), *G9* (*G9a*-null), *Gl* (*Glp*-null,) *S1* (*Suv39h1*-null), and *S2* (*Suv39h2*-null).

Series 1 clones were generated from *Eset1*-conditional MEF cell, after the first round of selection for high Cre recombinase activity resulting in the isolation of the Eset25 clone, referred to as *E1c* (see above). *E1c* cells were transfected with two sgRNA/Cas9 vectors targeting *Suv39h1* and *Suv39h2* and clone B1 (*E1c S1S2*) was selected as described above. This clone was further co-transfected with vectors targeting *Eset2*, *G9a* and *Glp*, and clone B1A6 (*E1cE2 S1S2 G9Gl*) was selected after western blotting (G9a, Glp) and exon sequencing (*Eset2*).

Series 2 was generated from clone A4 (*E1cE2*) described above. We first co-transfected vectors targeting *G9a* and *Glp* and selected clone E2 (*E1cE2 G9Gl*), which was subsequently transfected with vectors targeting *Suv39h1* and *Suv39h2* to generate clone E2F4 (*E1cE2 G9Gl S1S2*). After inducing *Eset1* deletion, cells lacking all 6 H3K9 KMTs are referred to as *6KO1* and *6KO2*.

### Cellular extracts and western blot analysis

Nuclear extracts from 10 million cells were prepared by cell lysis for 30 min at 4 °C in hypotonic buffer (10 mM HEPES pH 7.9, 5 mM MgCl_2_, 0.25 M sucrose, 0.1% NP-40) and protease inhibitors (Roche). Nuclei were isolated by centrifugation and washed in lysis buffer. Acid extraction of histones^46^ was performed in 0.4 N HCl at 4 °C overnight, followed by centrifugation to discard insoluble material and by neutralization with NaOH. For whole-cell extracts, cell pellets from 5 million cells were washed in PBS and lysed in RIPA buffer. SDS-PAGE analysis was conducted according to standard procedures, with antibodies against Gapdh (Santa Cruz, sc-3233, 1:5000 dilution), Setdb1 (Thermo Fisher, MA5-15722, 1:1000 dilution), G9a (Abcam, ab185050, 1:2500 dilution), Glp (Abcam, ab41969, 1:2500 dilution), Suv39h1 (Cell signaling, 8729, 1:2500 dilution), Suv39h2 (LSBio, LS-C116360, 1:2500 dilution), total H3 (Abcam, ab1791, 1:40000 dilution), H3K9me1 (Active Motif, 39681, 1:2500 dilution), H3K9me2 (Jenuwein Laboratory, #4677, 1:5000 dilution), H3K9me3 (Abcam, ab8898, 1:20000 dilution), H3 pan-acetyl (Millipore, 06-599, 1:1000 dilution), H4 pan-acetyl (Millipore, 06-866, 1:2500 dilution), H3K4me3 (Diagenode, C1540003, 1:1000 dilution), α-tubulin (Abcam, ab4074, 1:1000 dilution), or γH2A.X (Millipore 05-636, 1:750 dilution). As a positive control for γH2A.X detection, *E1c* MEFs were treated with Etoposide (Sigma E383), either for 3 h at 12.5 μM, or for 24 h at 1 μM.

### Histone extraction and nanoLC–MS analysis

After acid extraction of histones, histones samples were separated on 16% SDS Tris Glycine Wedge gels (Thermo Fisher) followed by colloidal coomassie staining (Instant Blue, Expedeon). Histone bands were excised and histone propionylation and subsequent protein digestion using trypsin (Promega) were performed as described^47^, with minor modifications. In-gel propionylation prior to trypsin digestion was conducted two times employing the method “H-42x”^47^ followed by the hydroxylamine-mediated reversal of overpropionylation. Peptide samples were desalted using STAGE tips^48^ and analyzed using LC-MS as previously described^49^ with minor modifications. Samples were separated within 60 min with a linear gradient from 2% buffer B (80% acetonitril in 0.1% formic acid) to 60% buffer B at a flow rate of 250 nl/min, and this was followed by a washout step (80% buffer B, 5 min, 500 nl/min) and re-equilibration (2% buffer B, 5 min, 500 nl/min). Mass spectrometry was carried out using the “sensitive method” (data-dependent acquisition) on Orbitrap QExactive Plus (Thermo Fisher Scientific)^50^.

Mass spectrometry raw data were analyzed using Peaks Studio version 8.5 (Bioinformatics Solution Inc.). MS raw files were searched using the Peaks de novo search algorithm and Peaks DB search against *Mus musculus* (mouse) UniprotKB protein sequence database (December 2015). Trypsin was selected as a cleaving enzyme, while a maximum of four missed cleavages, a minimum of six amino acids per peptide, 10 ppm MS peptide ion mass tolerance, and 0.2 Da MS/MS tolerance were allowed. Carbamidomethylation of cysteine was set as a fixed modification, whereas the following modifications were set as variable modifications: acetylation at the protein N terminus and at lysine residues, deamidation of asparagine or glutamine, oxidation of methionine, propionylation of lysine and mono-methylated lysine residues, mono-methylation of lysine and arginine residues, dimethylation of lysine and arginine residues, trimethylation of lysine residues, and phosphorylation of serine, threonine, and tyrosine. Maximum (allowed) variable PTMs per peptide were set to 4. The peptide and protein false discovery rate (FDR) was set to a maximum of 0.01 using the decoy-fusion database search strategy implemented in Peaks Studio.

Histone peptide intensities were exported and the sum of all peptide intensities (or features), belonging to each particular histone PTM (hPTM) mark, was calculated for histone H3 and histone H4 in each individual biological sample. This was followed by an inter-sample normalization to adjust the total intensity of all hPTMs to the same level in each biological replicate in each condition. To achieve this normalization, the summed peptide intensity of each particular hPTM was divided by the total intensity of a non-modified histone peptide, a histone peptide that is consistently identified and bears no hPTM mark throughout the whole experiment. Peptide “YRPGTVALR” and peptide “VFLENVIR” were used for normalizing mouse histone H3 (sequence: 41–49) and mouse histone H4 (sequence: 60–67), respectively.

Data were plotted using GraphPad Prism version 8.3.0.

### DNA methylation analysis

Genomic DNA was purified by phenol/chloroform extraction and ethanol precipitation according to standard procedures. DNA dot blot for 5mC was performed according to standard procedures^51^. DNA was spotted on nitrocellulose (Biorad) in 2× SSC and UV-cross-linked at 254 nm, 120 mJ/cm^2^. Membranes were probed with anti-5-methylcytosine antibody (Zymo research, clone 10G4, diluted 1:3000 in 3% BSA/PBST). For MS analysis of 5mC and 5hmC levels, genomic DNA was digested to nucleosides using Nucleoside Digestion mix (NEB, M0649S). Targeted nucleoside quantification by LC-MS was carried out using an Agilent 1290 Infinity II UHPLC inline with an Agilent 6495 QQQ-MS operating in MRM mode. MRM settings were optimized separately for unmodified nucleosides using pure standards and adapted for methylated nucleosides. LC separation was on a Waters T3 column (100 × 2.1 mm, 1.8-μm particles) using a solvent gradient of 100% buffer A (0.1% formic acid in water) to 90% buffer B (50:50 acetonitrile:methanol). Flow rate was 400 μL/min. Autosampler temperature was 4 °C, and the injection volume was 2 μL. Data processing was performed using Agilent MassHunter Software (version B.08).

### Indirect immunofluorescence and DNA FISH

Cells were grown on glass coverslips, washed with PBS, fixed at room temperature with 2% PFA for 10 min, and permeabilized with 0.5% Triton X-100 for 5 min followed by PBS washes. Immunofluorescence was performed with antibodies against H3K9me3 (Jenuwein Laboratory, #1926, 1:1000 dilution in 10% FBS/PBS), Lamin B (Santa Cruz, sc-6217, 1:200 dilution), HP1α (Millipore, 05-689, 1:500 dilution), Fibrillarin (Thermo Fisher, MA3-16771, 1:500 dilution), and Hmga1 (Abcam, ab129153, 1:500 dilution) for 1 h at room temperature. For DNA FISH, cells were dehydrated in ethanol. Denaturation at 80 °C for 5 min and hybridization at 37 °C for 1 h were performed in hybridization solution (2× SSC pH 7, 50 mM sodium phosphate pH 7, 60% formamide) with a mix of 4 LNA probes (3 nM each). Probe sequences are listed in Supplementary Table 1. Image acquisition was performed on an LSM 780 microscope (Zeiss) using ZEN2 black edition software (Zeiss).

### RNA sequencing

Total RNA was extracted with Trizol (Invitrogen), and the remaining DNA was digested with Turbo DNase (Ambion), followed by cleanup with RNeasy MinElute Cleanup kit (Qiagen, 74204). Total RNA libraries were prepared using the TruSeq Stranded Total RNA Library Prep Gold (Illumina, 20020598). Paired-end, 75 bp reads were generated with Illumina HiSeq3000 sequencer with a depth of 50 million reads per sample. Three biological replicates were sequenced per cell line.

### RNA-seq analysis

Unprocessed reads were first quality and adapter trimmed using cutadapt^52^ version 1.8.1 using a quality threshold of 20. Reads were then aligned to the GRCm38 genome from Ensembl using STAR^53^ version 2.5.2b, with settings as recommended by TEtranscripts^54^. Both repeats and genes were then quantified using TEtranscripts version 2.0.3 and differentially expressed repeat elements and genes determined using DESeq2^55^. For analysis of repeat element expression, library size factors as computed from the gene expression data were used rather than recomputing these using the expression metrics of the repeat elements themselves. BigWig files using in visualization tracks were constructed using deepTools^56^. For pathway analyses, normalized counts as computed by DESeq2, as well as fold changes and adjusted P values, were loaded into Ingenuity Pathway Analysis and the results subsequently plotted in R.

For visualization of coverage over repeat regions (Fig. 3c) reads where aligned to the repeat sequence extracted from Repbase^57^ using bwa mem^58^ version 0.7.17 with default settings, bigWig tracks were constructed using deepTools and IGV^59^.

### ATAC sequencing

For ATAC library preparation^60^, 50,000 cells were lysed in cold lysis buffer (10 mM Tris-HCl pH 7.4, 10 mM NaCl, 3 mM MgCl_2_, 0.1% v/v Igepal CA-630) and centrifuged at 4 °C for 10 min at 500*g*. The pellet was resuspended in a transposition reaction mixture provided by the Nextera DNA Library Prep kit (Illumina, 15028212) and incubated at 37 °C for 30 min. After transposition, the samples were purified with a Qiagen MinElute PCR purification kit (28604) according to the manufacturer’s instructions. For library preparation, each sample was subjected to PCR amplification with a pair of barcoded primers provided by the Nextera Index kit (Illumina 15055289). The reaction was monitored by qPCR and further cycles were performed when necessary, based on the amplification plot. Paired-end, 75 bp reads were generated with Illumina HiSeq3000 sequencer with a depth of 50 million reads per sample. Two biological replicates were sequenced per cell line.

### ATAC-seq analysis

Unprocessed reads were first quality and adapter trimmed using fastp^61^ version 0.19.4, using a mean window quality threshold of 3 and excluding low-complexity sequences. These were then aligned to the GRCm38 genome from Ensembl using STAR^53^ version 2.6.0c allowing no spliced alignments and with other options as described by TEtranscripts^54^. For extraction of the open-chromatin subset of reads, deepTools^56^ version 3.1.2 was used with a fragment length threshold of 150 bases. Alignments were subsequently quantified across repeats using TEtranscripts version 2.0.3 and the repeatmasker file from UCSC as the source for repeat locations. For visualization, bigWig tracks were constructed using deepTools and IGV^59^. Differential accessibility was determined using DESeq2^55^ version 1.20.0. Counts across gene bodies for genes showing unchanged expression in the RNA-seq data were used to determine sample-specific size factors, as other normalization methods would wash away global accessibility shifts. Pathway analysis was performed as with the RNA-seq data.

### Chromatin immunoprecipitation sequencing (ChIP-seq)

Ten million cells were trypsinized, washed with PBS, and cross-linked in 1% formaldehyde for 10 min at room temperature. After quenching with glycine (0.125 M), cells were pelleted, washed with cold PBS, and lysed in 1% SDS, 10 mM EDTA, 50 mM Tris-HCl, pH 8, and protease inhibitors (Roche). Lysates were sonicated (Covaris S220) to fragment sizes of 200–500 bp. For each ChIP, chromatin corresponding to 10 μg of DNA was diluted in 1% Triton X-100, 2 mM EDTA, 167 mM NaCl, 20 mM Tris-HCl, pH 8, and protease inhibitors, and incubated overnight at 4 °C with 4 μg of antibody against H3K9me1 (Abcam, ab8896), H3K9me2 (Abcam, ab1220) or H3K9me3 (Abcam, ab8898), followed by 4 h at 4 °C with 25 μl of Protein G dynabeads (Invitrogen). Beads were washed three times in low salt (150 mM NaCl) and once in high salt (500 mM NaCl) before elution in 1% SDS, 50 mM NaHCO_3_, RNAse A and Proteinase K treatment, and DNA purification with Qiagen MinElute PCR purification kit (28604). Enrichment was validated by qPCR for control loci (Supplementary Fig. 3A: Major satellite repeats, L1MdA 5’UTR, IAP-EY3, Zinc finger protein 180 and Oct4 promoter as positive controls, as well as Actin B promoter as a negative control). Primer sequences are listed in Supplementary Table 1. Libraries were prepared with NEBNext Ultra II DNA Library Prep Kit for Illumina (NEB, E7645L). Paired-end, 100 bp reads were generated with Illumina NovaSeq 6000 with a depth of 100 million reads per sample. Two biological replicates were sequenced per ChIP as well as for input material.

### ChiP-seq analysis

The quality of raw Fastq reads was checked using FastQC (http://www.bioinformatics.babraham.ac.uk/projects/fastqc). Reads were aligned to mm10 (GRCm38) with bwa version 0.7.1.^58^. Peaks were called using histoneHMM^62^. Peaks were annotated using ChiPseeker software^63^ version 1.18.0. Peaks that are located within + /− 1 kb of the TSS were extracted for downstream analysis. Heatmaps and profiles were generated using deeptools version 3.5.0^56^. Marks were aligned around the center of the peak (computeMatrix reference-point). Flanking regions were added to show the signal extending outside enriched regions. ComputeMatrix was executed with the following parameters:—skipZeros—binSize 10 -a 10000 -b 10000—referencePoint center. Heatmaps were generated using plotHeatmap. Coverage and normalization to the input were obtained using deepTools-3.5.0 bamCompare. FRiPs scores were obtained using deepTools/plotEnrichment. Violin plots represent the distribution of gene expression levels for each H3K9 methylation state. Normalized values of RNA-seq expression were obtained using DESeq2 version 1.26.0^55^ on the count matrices output from snakePipes version 2.1.0^64^. Red dots represent the mean expression level. Data visualization was performed using ggplot2^65^ version 3.3.2. *P* values comparing the means were calculated using the *t* test.

### Transmission electron microscopy (TEM) sample preparation and imaging

Cells grown on Thermanox coverslips (Nunc) were prepared as described^66^ and adapted for cell monolayers. Cells were fixed with 2.5% glutaraldehyde and 2% paraformaldehyde (Electron Microscopy Sciences EMS, 16400 and 15700) in 0.1 M Hepes buffer pH 7.4 for 1 h at room temperature and then overnight at 4 °C. After washes in cold 0.15 M cacodylate buffer pH 7.4 (Sigma Aldrich, CO250) the cells were post-fixed in 1.5% potassium ferrocyanide (Sigma Aldrich, 60280) and 2% osmium tetroxide (EMS, 19110) in 0.15 M cacodylate buffer pH 7.4 on ice for 30 min. The cells were then washed with double-distilled water (ddH2O) and immersed in filtered 1% thiocarbohydrazide (TCH, EMS, 21900) solution for 10 min. After this step, the cells were stained in 2% osmium tetroxide for 30 min, rinsed again in ddH_2_O, and stained with 1% uranyl acetate at 4 °C overnight. After washes with ddH_2_O, cells were stained with Walton’s lead aspartate (Sigma A9256 and EMS 17900) solution at 60 °C for 30 min and dehydrated in a graded alcohol series. After flat‐embedding in Epoxy resin (SERVA Electrophoresis GmbH 21045) samples were cured at 60 °C for 12 h. Coverslips were detached from the resin using repetitive freeze−thaw in liquid nitrogen. A region of interest containing cells was selected (~1 × 1 mm), cut, and mounted on an aluminum pin. For transmission electron microscopy (TEM), serial thin sections (60 nm) were cut using an ultramicrotome (Ultracut 7, Leica, Vienna) and imaged using a Spirit 120 kV (FEI, now THF, Eindhoven) at magnifications between 1700× and 8200× equipped with a side-mounted camera (Veleta, EMSIS Gmbh).

## Supplementary information

Supplementary Information

## Data Availability

The proteomics datasets generated in this study are available at ProteomeXchange via the PRIDE partner repository with record number PXD018175. The UniProtKB database is accessible at https://ftp.uniprot.org/pub/databases/uniprot/previous_major_releases/release-2015_12/knowledgebase/. A database filtered for mouse peptides is available as a FASTA file at ProteomeXchange, together with the datasets generated in this study. The RNA-seq, ATAC-seq, and ChIP-seq datasets generated in this study are available in the GEO repository with record number GSE142105. The Ingenuity Pathway Analysis (IPA) database is accessible at https://digitalinsights.qiagen.com/products-overview/discovery-insights-portfolio/analysis-and-visualization/qiagen-ipa/. The data and any unique materials are available from the corresponding author upon reasonable request. Source data are provided with this paper.

## Source data

Source Data
