# Supplementary material for: Complete loss of H3K9 methylation dissolves mouse heterochromatin organization
Source: Nat Commun. 2021 Jul 16;12:4359. doi: 10.1038/s41467-021-24532-8 (PMC8285382; doi:10.1038/s41467-021-24532-8)
Supplement: Supplementary file 1 — Supplementary Information [file 41467_2021_24532_MOESM1_ESM.pdf]

Supplementary Information

Complete loss of H3K9 methylation dissolves mouse heterochromatin organization

Thomas Montavon[1], Nicholas Shukeir[1], Galina Erikson[1], Bettina Engist[1], Megumi Onishi-Seebacher[1, #], Devon Ryan[1,&], Yaarub Musa[1,§], Gerhard Mittler[1], Alexandra Graff Meyer[2], Christel Genoud[2] and Thomas Jenuwein[1,*]

[1]Max-Planck Institute of Immunobiology and Epigenetics, Freiburg, Germany
[2]Friedrich Miescher Institute for Biomedical Research, Basel, Switzerland

[*]corresponding author: jenuwein@ie-freiburg.mpg.de

Present addresses:
[#] Novartis Institute for Biomedical Research (NIBR), Basel, Switzerland
[&] Genedata AG, Basel, Switzerland
[§] Thermo Fisher Scientific GmbH, Dreieich, Germany

Includes 5 Figures and 1 Table.

Supplementary Figure 1

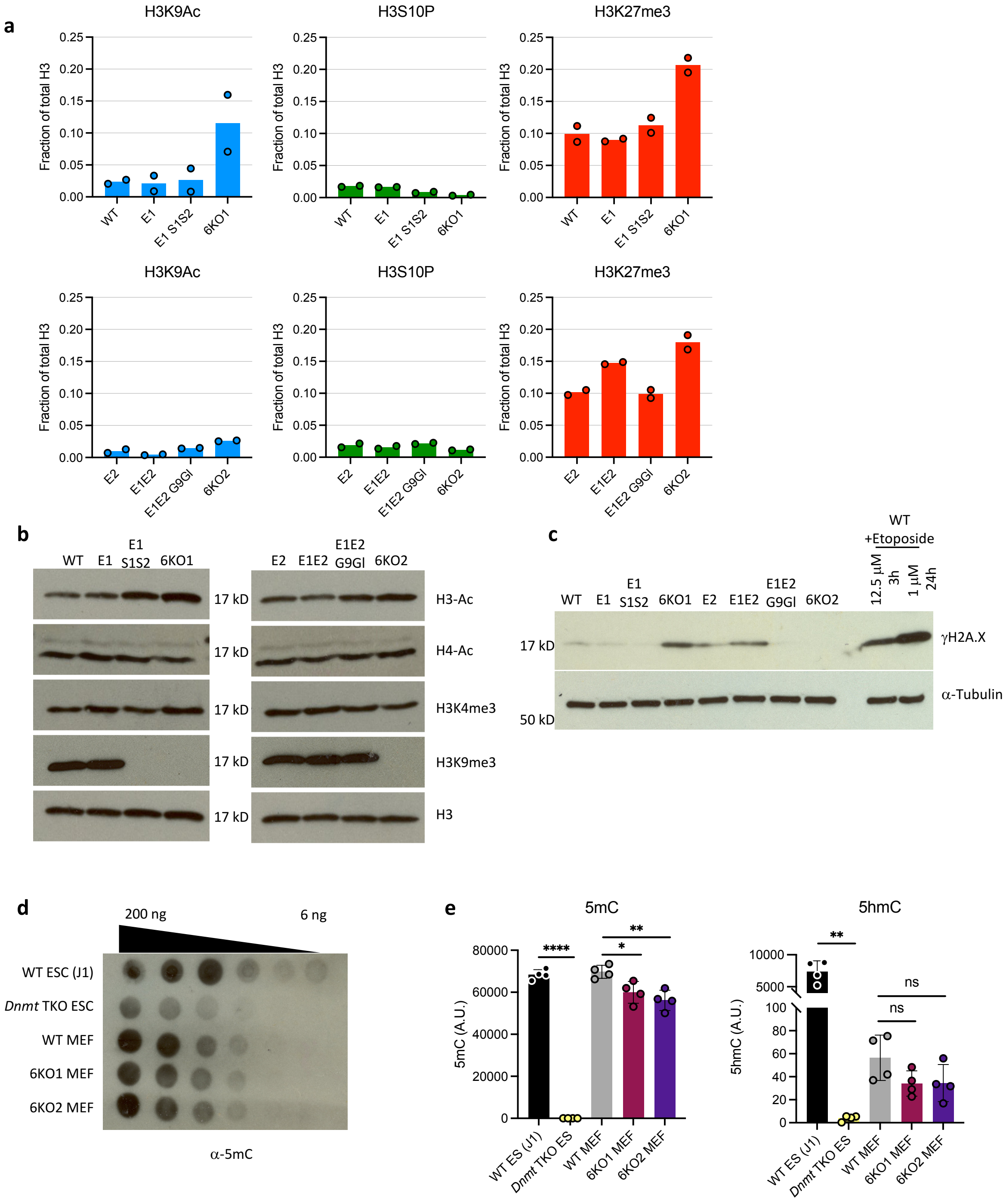

Supplementary Figure 1. Histone modifications and DNA methylation in H3K9 KMT mutant MEF cells.

(a) Mass spectrometry analysis of H3K9ac, H3S10P, and H3K27me3 in acid-extracted histones from compound H3K9 KMT mutants. The y-axis indicates the fraction of total H3 carrying the indicated histone modification, determined by normalizing intensities obtained for the corresponding peptides in nanoLC-MS (bars represent mean and individual data points are overlaid, N=2 independent samples).

(b) Western blot analysis of histone modifications in compound H3K9 KMT mutants. Acid-extracted histones were probed with antibodies specific for pan-acetyl H3, pan-acetyl H4, H3K4me3 and H3K9me3. Total H3 was used as loading control. Representative results of 3 independent experiments. Source data are provided as a Source Data file.

(c) Western blot for γH2A.X in compound H3K9 KMT mutants. Whole-cell extracts were probed with an antibody specific for phosphorylated H2A.X (Ser139). Etoposide treatment of WT cells served as a positive induction of γH2A.X. αTubulin was used as a loading control. Representative results of 2 independent experiment. Source data are provided as a Source Data file.

(d) DNA dot-blot analysis for 5-metylcytosine (5mC) levels in genomic DNA from MEFs lacking all 6 SET domain H3K9 KMT (*6KO1* and *6KO2*). Serial dilutions of genomic DNA were probed with an antibody specific for 5mC. Wild type and *Dnmt* triple knock-out (*Dnmt* TKO) ES cells were included as controls.

(e) Mass spectrometry analysis of DNA methylation levels. Total genomic DNA was digested to nucleosides and subjected to LC-MS. Relative abundances of 5mC and 5-hydroxymethyl cytosine (5hmC) are shown on the y-axis (mean ± S.D., individual data points overlaid, N=4 independent samples). Wild type and *Dnmt* TKO ES cells were included as controls. Asterisks indicate statistically significant differences when compared to *WT* levels (5mC: *, $P = 0.024$ for *6KO1* vs *WT* MEF; **, $P = 0.005$ for *6KO2* vs *WT* MEF; ****, $P = 1.2 \times 10^{-5}$ for *Dnmt* TKO vs *WT* ES; 5hmC, **, $P = 0.003$ for *Dnmt* TKO vs *WT* ES; two-sided, unpaired t-test).

Supplementary Figure 2

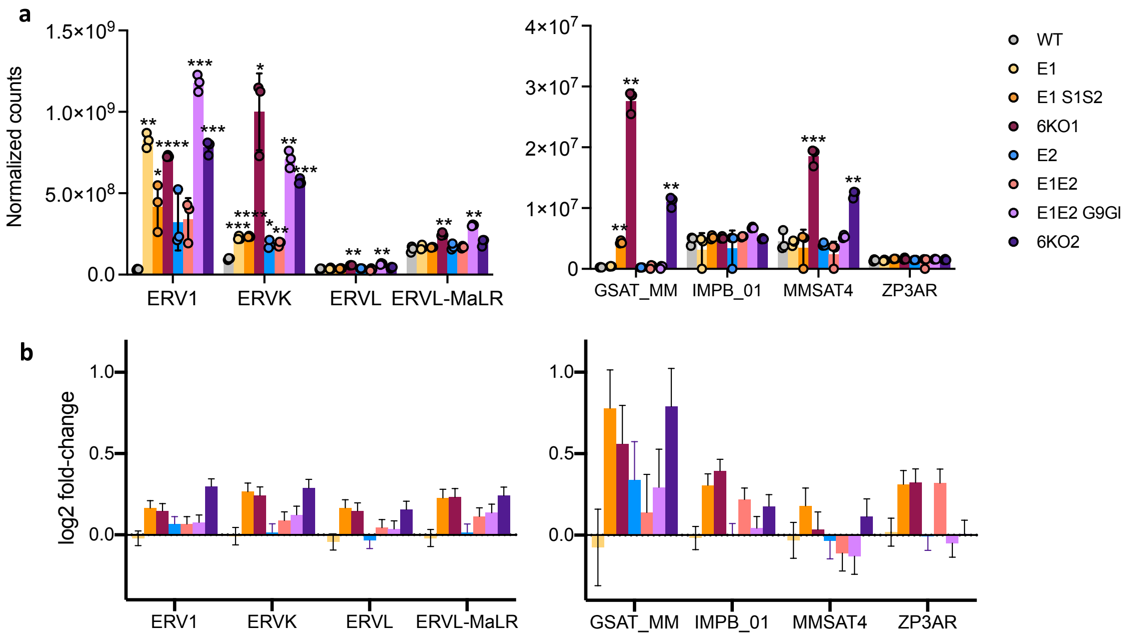

Supplementary Figure 2. Derepression of distinct repeat families in H3K9 KMT mutant MEF cells.

(a) Global changes in expression for specific families of LTR (ERV1, ERVK, ERVL and ERVL-MaLR, left graph) or satellite (GSAT_MM, IMPB_01, MMSAT4 and ZP3AR, right) repeats in compound H3K9 KMT mutants. The y-axis indicates normalized read counts (mean ± S.D., individual data points overlaid, N=3 independent samples). Asterisks indicate statistically significant differences when compared to WT levels (ERV1: **, $P = 1.0 \times 10^{-3}$ for *E1*, *, $P = 0.043$ for *E1 S1S2*, ****, $P = 1.3 \times 10^{-7}$ for *6KO1,* ***, $P = 6.7 \times 10^{-4}$ for *E1E2 G9Gl,* ****, $P = 9.9 \times 10^{-4}$ for *6KO2*; ERVK: ***, $P = 4.1 \times 10^{-4}$ for *E1*, ****, $P = 1.2 \times 10^{-5}$ for *E1 S1S2*, *, $P = 0.02$ for *6KO1* and for *E2*, **, $P = 4.6 \times 10^{-3}$ for *E1E2*, **, $P = 2.3 \times 10^{-3}$ for *E1E2 G9Gl,* ***, $P = 1.4 \times 10^{-4}$ for *6KO2*; ERVL: **, $P = 4.4 \times 10^{-3}$ for *6KO1* and $P = 1.1 \times 10^{-3}$ for *E1E2 G9Gl;* ERVL-MalR: **, $P = 3.3 \times 10^{-3}$ for *6KO1* and $P = 2.7 \times 10^{-3}$ for *E1E2 G9Gl;* GSAT_MM: **, $P = 9.3 \times 10^{-3}$ for *E1 S1S2,* $P = 1.6 \times 10^{-3}$ for *6KO1,* $P = 2.2 \times 10^{-3}$ for *6KO2*; MMSAT4: ***, $P = 3.8 \times 10^{-4}$ for *6KO1, **,* $P = 7.6 \times 10^{-3}$ for *6KO2,* two-sided, unpaired t-test). (b) Change in DNA accessibility for specific families of LTR and satellite repeats in compound H3K9 KMT mutants. The y-axis indicates log2 fold change of normalized ATAC-seq reads for compared to WT (mean ± S.D., N=2 independent samples).

Supplementary Figure 3

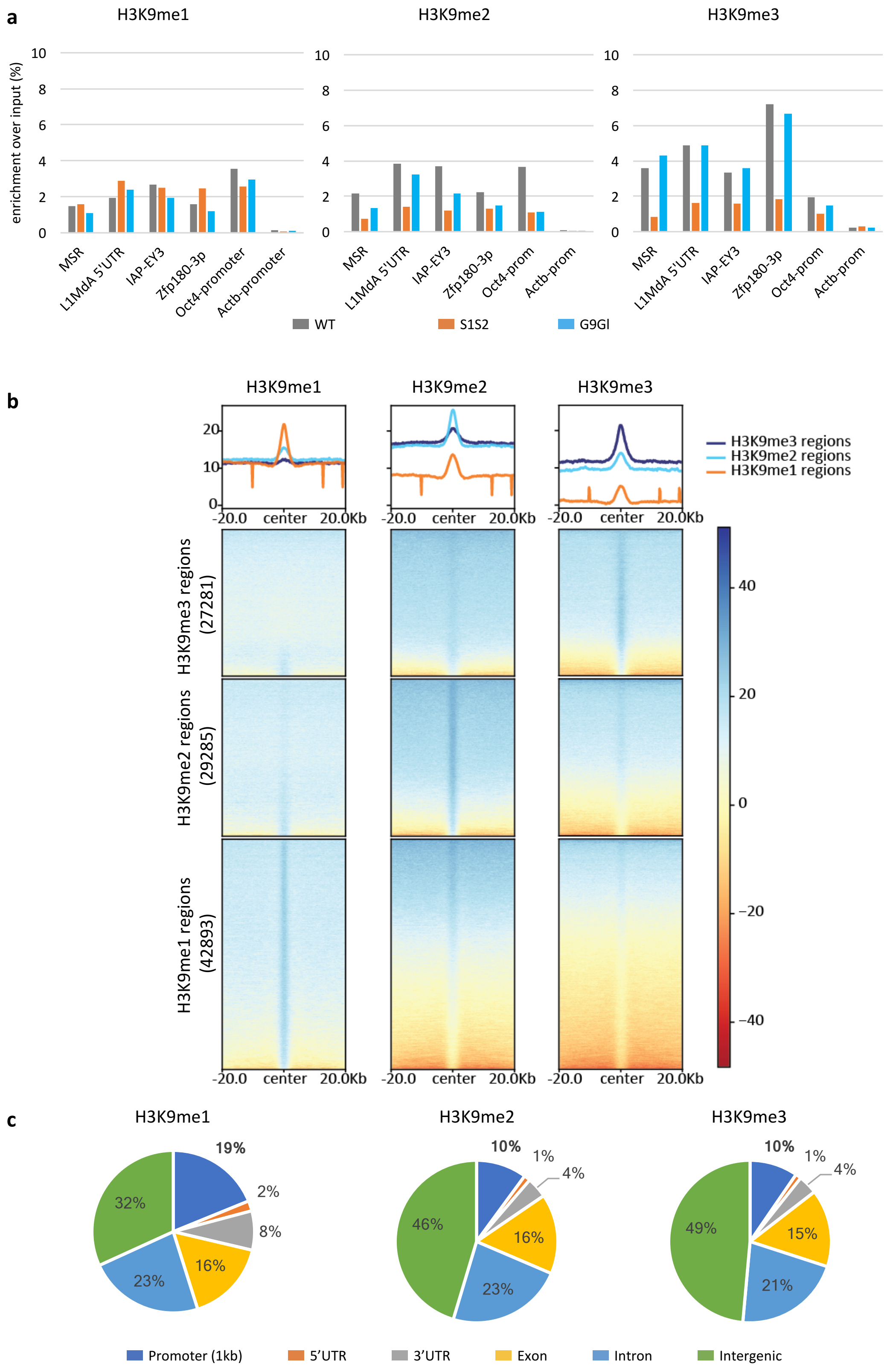

Supplementary Figure 3. Genome-wide mapping of H3K9me1, H3K9me2 and H3K9me3 in *Eset1cKO* MEF cells.

(a) Validation of H3K9 methyl antibodies used for chromatin immunoprecipitation (ChIP-qPCR). Enrichment over input is shown for selected loci known to harbour H3K9 methylation: Major satellite repeats (MSR), L1MdA 5'UTR, IAP-EY3, Zinc finger protein 180 (Zfp180), POU domain class 5 transcription factor 1 (Oct4). In addition, one negative control, Actin b promoter, is shown. Antibodies were tested on chromatin isolated from WT MEF cells (grey), as well as from *Suv39h1/Suv39h2* (*S1S2*, orange) and *G9a/Glp* (*G9Gl*, blue) mutants. N = 1 experiment.

(b) Comparative heatmaps for H3K9me1 (left), H3K9me2 (middle) and H3K9me3 (right) distribution. Heatmaps extend +/- 20 kb from the center of the enriched regions.

(c) Pie charts showing distribution of H3K9me1 (left), H3K9me2 (middle) and H3K9me3 (right) methylated regions over genomic features (promoters, 5' and 3'UTR, exons, introns and intergenic regions). Promoter regions (bold numbers) are defined as sequences within +/-1 kb of the transcription start site (TSS).

Supplementary Figure 4

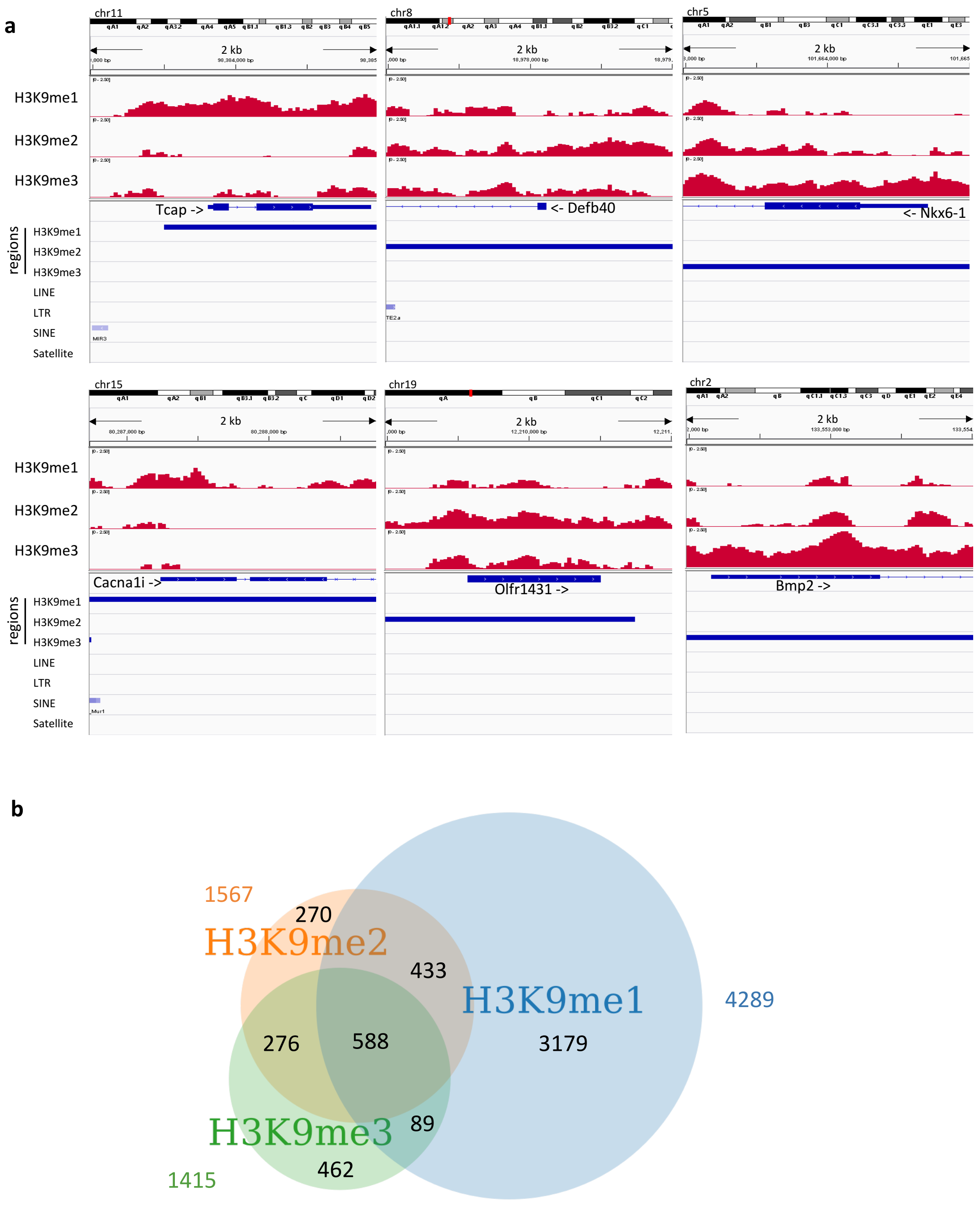

Supplementary Figure 4. H3K9 methylation at gene promoters.

(a) Genome browser tracks showing H3K9me1, H3K9me2 or H3K9me3 enrichment over input (log2 fold change, red) at example gene promoters, as determined by ChIP-seq in WT MEFs. Two example promoters are shown for each H3K9 methylation state: H3K9me1, Titin-cap (*Tcap*) and Calcium channel, voltage-dependent, alpha 1I subunit (*Cacna1a*); H3K9me2, Defensin beta 40 (*Defb40*) and Olfactory receptor 1431 (*Olfr1431*); H3K9me3, NK6 homeobox 1(*Nkx6-1*) and Bone morphogenetic protein 2 (*Bmp2*). Each browser track shows a 2 kb genomic interval surrounding the TSS. Positive peak is indicated by blue bars below gene annotation.

(b) Venn diagram of H3K9 methylated gene promoters. The total numbers of genes decorated by H3K9me1, H3K9me2, or H3K9me3 over their promoter (+/- 1 kb of the TSS) is indicated beside each colored area. Overlaps indicate genes with promoters that are positive for more than one H3K9 methylation state.

Supplementary Figure 5

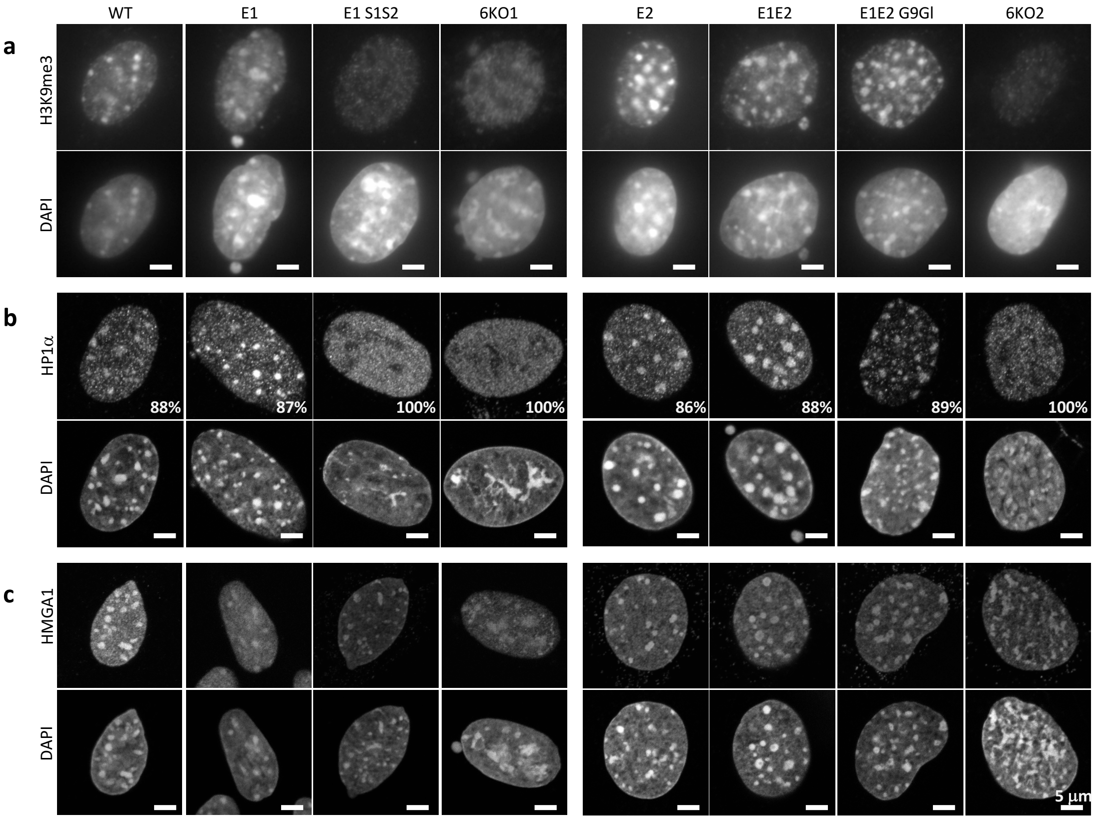

Supplementary Figure 5. Heterochromatin markers in H3K9 KMT mutant MEF cells.

(a) Immunofluorescence for H3K9me3. Representative images (N=100) are shown. For each image, lower panels show DAPI counterstaining of the same nuclei.

(b) Immunofluorescence for HP1α. Percentages indicate cells that display the shown pattern. N= 203 cells examined for WT, 157 for *E1*, 214 for *E1 S1S2*, 101 for *6KO1,* 194 for *E2*, 114 for *E1E2*, 122 for *E1E2 G9Gl* and 103 for *6KO2*. For each image, lower panels show DAPI counterstaining of the same nuclei.

(c) Immunofluorescence for HMGA1. Representative images (N=200) are shown. For each image, lower panels show DAPI counterstaining of the same nuclei. Scale bars represent 5 micrometers (μm).

Supplementary Table 1. List of oligonucleotide sequences.

Primer name	Type	Target locus	Sequence
Setdb2_ex7_gRNA	gRNA	Setdb2 exon7	GTTTCGTAGATTCCGTCCAC
Setdb2_ex8_gRNA	gRNA	Setdb2 exon8	CATTCTCCATCCGATGACAA
Suv39h1_gRNA1	gRNA	Suv39h1 exon3	AGCTTGTCCGACGACACCGC
Suv39h1_gRNA2	gRNA	Suv39h1 exon3	GCGGGAGTTGCACTCGTAGA
Suv39h2_gRNA1	gRNA	Suv39h2 exon3	TCTACTACATTAACGAGTAC
Suv39h2_gRNA2	gRNA	Suv39h2 exon3	TGCAACTCAAGGTGTCGATG
G9a_ex21_gRNA	gRNA	G9a exon21	TACTTGTAGTCCTCCGGGCA
G9a_ex22_gRNA	gRNA	G9a exon22	GTCAGCTCAGTATCCGATGC
Glp_ex21_gRNA	gRNA	Glp exon21	GAGACATACTTATAGTTGGT
Glp_ex23_gRNA	gRNA	Glp exon23	GAGGAACTGCCGCAATCGTG
Setdb2_ex7F	Surveyor	Setdb2 exon7	TTCCATCGCCATATCTGCTC
Setdb2_ex7R	Surveyor	Setdb2 exon7	TCGGGGCCATACTGTATTTCTA
Setdb2_ex8F	Surveyor	Setdb2 exon8	AGCCTGTGGTTGGTGTGATT
Setdb2_ex8R	Surveyor	Setdb2 exon8	TGAAACCTGTAAGACTGTAGACCA
Suv39h1_F1	Surveyor	Suv39h1 exon3	CCTGGCTGGTCAGCAATGTT
Suv39h1_R1	Surveyor	Suv39h1 exon3	GTGGGTGCCAACAGACAGT
Suv39h1_F2	Surveyor	Suv39h1 exon3	GGAGCCACGGCAGAATCTAAA
Suv39h1_R2	Surveyor	Suv39h1 exon3	GGGTTAGGGGCACACATACTAC
Suv39h2_F1	Surveyor	Suv39h2 exon3	TGGCCAGATTCTACAAACACCT
Suv39h2_R1	Surveyor	Suv39h2 exon3	TGCCAATGAACCCTTCTCCT
Suv39h2_F2	Surveyor	Suv39h2 exon3	GGCCAGATTCTACAAACACCTG
Suv39h2_R2	Surveyor	Suv39h2 exon3	ATGCCAATGAACCCTTCTCC
G9a_ex21-F	Surveyor	G9a exon21	ACTAGAGCGCGCATTTGCAT
G9a_ex21-R	Surveyor	G9a exon21	TAGCCGAGATTGTTCTCCGTGG
G9a_ex22-F	Surveyor	G9a exon22	TTAAACTCCTGGAGCAGCCG
G9a_ex22-R	Surveyor	G9a exon22	GAACTGCGAGACATCGACCA
Glp_ex21-F	Surveyor	Glp exon21	TTGGCCTTTATTTTCCCTTTTAGC
Glp_ex21-R	Surveyor	Glp exon21	GCTTAGACCAACATGGACCCTC
Glp_ex23-F	Surveyor	Glp exon22	CAGTCAGTAGCCTTGGGACC
Glp_ex23-R	Surveyor	Glp exon22	ACAGGAGGCACAGGAAAAGG
Act-b-F	ChIP-qPCR	Actb promoter	AGCCAACTTTACGCCTAGCGT
Act-b-R	ChIP-qPCR	Actb promoter	TCTCAAGATGGACCTAATACGGC
Oct4-F	ChIP-qPCR	Oct4 promoter	CCAGTCACACCCAACCTCTT
Oct4-R	ChIP-qPCR	Oct4 promter	CTGGCCCTTGTCATGTAGGT
Zfp180-F	ChIP-qPCR	Zfp180 exon5	CCGTACAGGTGCAATCTGTG
Zfp180-R	ChIP-qPCR	Zfp180 exon5	GTTTGTAGCTCTGGCGGAAC
MSR-F	ChIP-qPCR	MSR	TGGAATATGGCGAGAAAACTG
MSR-R	ChIP-qPCR	MSR	AGGTCCTTCAGTGGGCATTT
L1MdA-F	ChIP-qPCR	L1MdA 5'UTR	ACTGCGGTACATAGGGAAGC
L1MdA-R	ChIP-qPCR	L1MdA 5'UTR	TGTGATCCACTCACCAGAGG
IAP-EY3-F	ChIP-qPCR	IAP-EY3	ACAGAGGAGGACAACTGCTC
IAP-EY3-R	ChIP-qPCR	IAP-EY3	AACCTTACACAGGCAAAAGC
MSR-probe1	DNA FISH	MSR	aTtTaGaAaTgTcCaCtGtAgGaC
MSR-probe2	DNA FISH	MSR	aAtAtGgCaAgAaAaCtGaAaAT
MSR-probe3	DNA FISH	MSR	aAtGaGaAaCaTcCaCtTgAcGaCT
MSR-probe4	DNA FISH	MSR	tGaAaAaTgAgAaAtGcAcAcTG

For DNA Fish probes, lowercase indicates locked nucleic acid.
